# Genome-wide association meta-analysis and rare copy number variant analysis of treatment-resistant depression

**DOI:** 10.1038/s41380-025-03084-z

**Published:** 2025-06-26

**Authors:** Ying Xiong, Kristi Krebs, Bradley Jermy, Robert Karlsson, Joëlle A. Pasman, Thuy-Dung Nguyen, Tong Gong, Kaarina Kowalec, Christian Rück, Robert Sigström, Lina Jonsson, Caitlin C. Clements, Elin Hörbeck, Evelyn Andersson, Julia Bäckman, FinnGen, Estonian Biobank Research Team, Andrea Ganna, Jakob German, Patrick F. Sullivan, Mikael Landén, Kelli Lehto, Yi Lu

**Affiliations:** 1 Department of Medical Epidemiology and Biostatistics, Karolinska Institutet, Stockholm, Sweden; 2 Estonian Genome Centre, Institute of Genomics, University of Tartu, Tartu, Estonia; 3 Institute for Molecular Medicine Finland, FIMM, HiLIFE, University of Helsinki, Helsinki, Finland; 4 College of Pharmacy, University of Manitoba, Winnipeg, MB Canada; 5 Centre for Psychiatry Research, Department of Clinical Neuroscience, Karolinska Institutet, Stockholm, Sweden; Stockholm Health Care Services, Region Stockholm, Stockholm, Sweden; 6 Department of Psychiatry and Neurochemistry, Institute of Neuroscience and Physiology, Sahlgrenska Academy, University of Gothenburg, Gothenburg, Sweden; 7 Department of Affective Disorders, Sahlgrenska University Hospital, Gothenburg, Sweden; 8 Department of Psychology, University of Notre Dame, South Bend, IN USA; 9 Analytic and Translational Genetics Unit, Department of Medicine, Massachusetts General Hospital, Boston, MA USA; 10 Eric and Wendy Schmidt Center, Broad Institute of MIT and Harvard, Cambridge, MA USA; 11 Departments of Genetics and Psychiatry, University of North Carolina at Chapel Hill, Chapel Hill, NC USA

**Keywords:** Genetics, Depression

## Abstract

Treatment-resistant depression (TRD), defined as major depressive disorder (MDD) with multiple failed responses to antidepressant treatments, has been suggested to be heritable, but identifying its genetic component is challenging. Using a restrictive TRD definition based on antidepressant medication followed by electroconvulsive therapy (ECT), which may represent a severe subset of TRD cases, we investigated both common variants and rare copy number variations (CNVs) associated with a) TRD risk (2 062 TRD vs. 441 037 healthy controls) and b) treatment resistance in MDD (2 062 TRD vs. 38 544 non-TRD) across three Nordic countries. We observed a significant SNP-based heritability for TRD risk at 26% (SE = 5%). Genome-wide association analysis identified one locus on chromosome 3 (intronic region of *SPATA16*) for TRD risk and one suggestive locus for treatment resistance in MDD. TRD risk showed positive genetic correlations (*r*_*g*_) with other psychiatric disorders, with notably *r*_*g*_ with bipolar disorder (0.86, SE = 0.20) and schizophrenia (0.57, SE = 0.13), as well as a negative *r*_*g*_ with intelligence (−0.13, SE = 0.07). Analyses using PRS showed that TRD had higher common-variant burdens of various psychiatric disorders compared to non-TRD. Furthermore, TRD carried a higher CNV deletion burden in total and average lengths than healthy controls or non-TRD cases and was associated with a group of 54 known neuropsychiatric CNVs (ORs = 1.74–2.86). Given that our definition of TRD involves the use of ECT, our findings may reflect a severe form of treatment resistance. This work adds evidence on a genetic basis and provides insights into the genetic architecture of TRD, underscoring the need for further genomic research into this ‘difficult-to-treat’ condition.

## Introduction

Major depressive disorder (MDD) is a leading cause of disability, imposing significant individual burden [1–3] and societal costs [4]. Roughly two-thirds of individuals with MDD do not achieve complete remission after an initial antidepressant trial, with some developing treatment-resistant depression (TRD) when failing to respond to multiple adequate treatment trials [5, 6]. TRD is associated with worse health outcomes, including higher risks of hospitalization, suicide, and mortality compared to other MDD cases, compounding the overall impact of MDD [7–10]. Unravelling the biological underpinnings of TRD is crucial for improving prognosis and lessening the substantial impact of the condition.

Treatment-related phenotypes are likely to have a heritable component. Both common and rare genetic variations contribute to the development of TRD [11]. To date, no pedigree-based heritability of TRD risk (estimated by comparing TRD and healthy controls) has been reported. While significant heritability estimates have been reported for treatment resistance in MDD (estimated by comparing TRD with non-TRD MDD cases), these estimates vary widely across different definitions [12]. The single nucleotide polymorphism (SNP)-based heritability (${h}_{{SNP}}^{2}$, capturing common genetic variants) ranged from 15–25% for TRD risk and 2–37% for treatment resistance in MDD [13–16]. Genome-wide association studies (GWAS) on TRD have identified five loci that require further replication [12–17]. Copy number variations (CNVs) have been investigated in neuropsychiatric disorders [18–21] and suggested to affect treatment response by disrupting drug metabolism or modulating the expression of drug targets [22, 23]. Previous research, however, found limited evidence for rare CNVs in TRD [23]. Other attempts, such as whole exome or whole genome sequencing, are promising, but no rare CNVs or rare variants have been identified for TRD [24, 25].

Limited genetic findings for TRD are likely associated with challenges in defining the phenotype [26–29] and collecting large samples [11]. Emerging research highlights the potential of using large biobanks to address these challenges by augmenting sample sizes with well-defined phenotypes based on patient treatment records [13]. In this study, we aimed to investigate the genetic underpinnings of TRD using biobanks and register data from three Nordic countries. Electroconvulsive therapy (ECT) is often used for severe MDD cases who do not respond to first- or second-line treatment. We, therefore, adopted a restrictive TRD definition based on both ECT and antidepressant use to capture a homogeneous and severe subset of TRD cases [30]. By comparing these TRD cases with healthy controls and non-TRD MDD cases in GWAS and CNV burden analyses, our study pursued four specific objectives: (1) to estimate the ${h}_{{SNP}}^{2}$ of TRD risk and treatment resistance in MDD; (2) to identify common genetic variants associated with TRD risk and treatment resistance in MDD; (3) to explore the genetic overlap between TRD risk or treatment resistance in MDD and related traits; (4) to examine the global burden of CNV and explore the role of known neuropsychiatric CNVs in TRD risk and treatment resistance in MDD.

## Methods

### Study samples

**Fig. 1:**
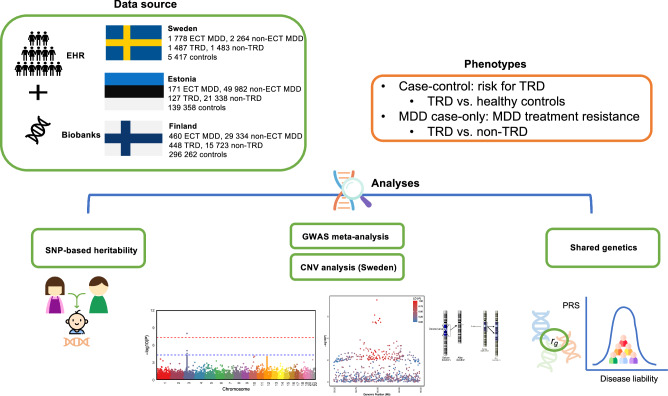
Overview of the study. Data sources, phenotypes, and analyses used in the study. First, we estimated the SNP-based heritability of TRD risk and treatment resistance in MDD in the Swedish cohort. Second, we conducted GWAS meta-analyses and examined shared genetics between TRD and other psychiatric disorders or cognitive traits. Last, we conducted CNV burden analyses and tested the association between neuropsychiatric CNVs and TRD in the Swedish cohort.

With the relatively low prevalence of ECT use, we maximized the sample size by combining ascertained cases from Sweden, and by extracting ECT records from register data linked with large biobanks including Estonian Biobank (EstBB) and FinnGen (Fig. 1).

#### Sweden

We combined samples from three Swedish cohorts: 1) the Predictors for ECT (PREFECT) study (*N* = 1 922) [31]; 2) The internet-based cognitive behavior therapy *(*iCBT) study (*N* = 964) [32, 33]; 3) Swedish Twin Studies of adults: Genes and Environment (STAGE) (*N* = 9 555) [34] and linked with the National register data.

#### EstBB

A population-based biobank with longitudinal records from the electronic health records (EHR) database and biological samples, linked with National primary and specialist care data. Genotyping and EHR data are available for about 200 000 individuals [35, 36].

#### FinnGen

A large-scale research project using samples collected by a network of Finnish biobanks with genotype data, linked with Finnish National health registries for EHR data. Genotype and health registry data are available for around 356 000 individuals [37].

Detailed descriptions of cohorts, and register data information including ECT codes, phenotype definition, genotyping, quality control, and imputation are in Supplementary Methods S1, Supplementary Table 1, and Supplementary Table 2.

### Ethics approval and consent to participate

The study across all cohorts was approved by the appropriate local institutional review boards (Details are in Supplementary Methods S1). The meta-analysis was conducted in Sweden and used summary-level data from Sweden, EstBB, and FinnGen. All methods in this study were performed in accordance with the relevant guidelines and regulations. Informed consent was obtained from all participants in the Swedish cohort, EstBB, and FinnGen as part of their respective cohort or biobank recruitment procedures.

### Phenotype definition

We studied TRD risk (TRD versus healthy controls) and treatment resistance in MDD (TRD versus non-TRD MDD cases). We defined TRD, non-TRD, and healthy controls, similarly to our previous study [30], following the definition criteria below (Details in Supplementary Methods S1–S2 and Supplementary Table 2).

#### TRD

As ECT can be used to treat MDD cases with antidepressant treatment failure, we used MDD diagnosis, antidepressant, and ECT records to define TRD. Among ECT-treated MDD cases, we selected MDD patients who received at least one antidepressant of adequate duration (>6 weeks) before their first ECT treatment (a proxy for “previous antidepressant use”) to avoid including MDD cases received ECT for psychotic symptoms or other life-threatening conditions who need rapid symptom improvement [38]. We defined the treatment episode for each antidepressant as the gap between two consecutive dispenses of the same antidepressant, allowing a maximum gap of 120 days. Treatment duration was calculated from the first to the last dispense date of the antidepressant. We applied these algorithms consistently across all study sites. In the Swedish samples, we had information on the indication for ECT and included only individuals receiving ECT for a major depressive episode in the context of MDD, excluding those treated for major depressive episodes in the context of bipolar disorder, schizoaffective disorder, or other mood disorders. In the EstBB and FinnGen samples, where ECT indication data were unavailable, we excluded individuals with any lifetime diagnosis of bipolar disorder, schizophrenia, or schizoaffective disorder from the ECT-treated MDD group. The detailed info to derive the treatment duration was described previously [30], with technical details of the derivation process for each study site provided in Supplementary Methods S1–S2. The related diagnosis codes, medication codes, and operation codes for ECT are listed in Supplementary Table 2.

#### Non-TRD

Non-TRD was defined as MDD patients without ECT treatment and having no more than two antidepressants with adequate duration (>6 weeks) to align with existing definitions in the literature. In the non-TRD cases group, individuals with diagnoses of schizophrenia, schizoaffective disorder, or bipolar disorders were further excluded across three study sites.

#### Healthy controls

Individuals without any lifetime diagnosis of MDD were categorized as healthy controls. We further excluded individuals without MDD diagnosis who had a history of schizophrenia, schizoaffective disorder, or bipolar disorders across all three study sites.

Since previous research has used ECT as a surrogate for TRD [16], we further conducted sensitivity analyses among ECT-treated MDD, non-ECT-treated MDD, and healthy controls (see Supplementary Methods S2 and Supplementary Table 2 for relevant definitions).

### Genotyping, quality control, and imputation

Genotyping and quality control (QC) for each cohort are described in Supplementary Methods S1. Each study site conducted comparable QC procedures to process the genotype data.

### CNV calling and QC

In addition to common genetic variants, we derived rare CNVs to examine the association between rare CNVs and TRD risk or treatment resistance in MDD. We focused on the CNV analysis on the largest sample (the combined Swedish cohort). We adapted the EnsembleCNV pipeline to derive CNVs from genotype data [39], which integrates PennCNV [40], QuantiSNP [41], and iPattern [42]. We required CNVs to be called by at least two CNV methods in each individual (Supplementary Methods S3 and Supplementary Table 3). We included samples of European ancestry passing GWAS QC.

### Statistical analysis

#### SNP-based heritability

We first estimated ${h}_{{SNP}}^{2}$ to evaluate the heritable component of TRD risk and treatment resistance in MDD. We focused on the Swedish cohort, given that the majority of TRD samples were collected in Sweden. Using the genome-wide complex trait analysis (GCTA) GREML-LDMS module, we estimated ${h}_{{SNP}}^{2}$ with high-quality common markers (minor allele frequency, MAF > 0.01 and INFO score > 0.9) from individual imputed genotype data, adjusting for the first five ancestry principal components (PCs) [43–45]. We then converted ${h}_{{SNP}}^{2}$ from the observed scale to the liability scale, accounting for extreme phenotype selection based on population prevalence assumptions [46]. We assumed that within MDD cases, the proportion of severe TRD treated by ECT was 10% and non-TRD was 70%; therefore, the population prevalence of severe (ECT-treated) TRD was 1.5% (assuming a prevalence of lifetime MDD of 15% and the proportion of severe (ECT-treated) TRD cases among MDD cases of 10%; 15% × 10% = 1.5%) [31]; and the prevalence of being a healthy control was 85% [47]. Given the uncertainty of these population prevalences, we also reported ${h}_{{SNP}}^{2}$ under a range of prevalences: 0.25–1.5% for TRD and 5–30% for treatment resistance in MDD.

#### GWAS meta-analysis

The details about the GWAS methods employed at each study site and annotations of GWAS meta-analysis results are described in Supplementary Methods S1 and S4. Manhattan plots, Q-Q plots, inflation factor, and linkage disequilibrium score regression (LDSC) [48] intercept for each GWAS were evaluated before the GWAS meta-analysis. We ran a meta-analysis using the inverse-variance weighted method with the fixed-effects model in METAL [49]. The heterogeneity of SNP effects was assessed using Cochran’s I^2^ statistics. Genomic inflation and population stratification effects were assessed using lambda (λ), lambda1000 (λ_1000_; accounts for case-control balance by scaling the λ value to 1000 cases and 1000 controls), and the LDSC intercept. The genome-wide significance and suggestive significance were set as *P* < 5.0 × 10^−8^ and *P* < 5.0 × 10^−5^, respectively. We reported ${h}_{{SNP}}^{2}$ estimated by LDSC using summary statistics of GWAS meta-analysis.

#### Genetic correlation

We ran LDSC to estimate genetic correlations (*r*_*g*_) between TRD and schizophrenia, bipolar disorder, anorexia nervosa, MDD, attention deficit hyperactivity disorder (ADHD), autism spectrum disorder (ASD), educational attainment, intelligence, and personality traits using the most recent GWAS summary statistics (Supplementary Table 4) [50–60]. A multiple comparison correction was applied using the false discovery rate (FDR < 0.05). We additionally compared the *r*_*g*_ of TRD with the *r*_*g*_ of MDD [53] using two-sample Z-tests to evaluate significant differences.

#### Polygenic risk scores

To further investigate genetic overlap with related traits, we conducted association analyses using polygenic risk score (PRS). We used SbayesR to rescale the summary statistics to account for linkage disequilibrium (LD) [61] (Supplementary Methods S5) and calculated PRS in PLINK2.0 [62]. We standardized scores at each site before running logistic regression to estimate odds ratios (OR) corresponding to per standard deviation (SD) increase in the PRS, adjusting for ancestry PCs. Finally, we conducted a fixed-effect meta-analysis to pool estimates using the inverse-variance weighted method (R package *Metafor*) [63].

Additionally, to investigate the impact of psychotic depression, we conducted a sensitivity analysis further excluding psychotic depression (with international classification code (ICD)-10 code F32.3 or F33.3) cases from the TRD group or ECT-treated MDD group.

#### CNV analysis

We conducted a global burden test and association test with known neuropsychiatric CNVs. In the global burden test, we examined the genome-wide CNV burden of TRD risk and treatment resistance in MDD. We derived three CNV features per individual using PLINK: the number of CNVs, the total length of CNVs, and the average length of CNVs. These CNV features are summarized for deletions and duplications separately. We tested the association between TRD and each CNV feature compared to healthy controls or non-TRD in logistic regressions. For each association test between CNV burden and TRD, we performed permutation tests with 10 000 rounds to assess the statistical significance by randomly swapping case-control status within the sample. The significance threshold was set at an empirical *P* value (*P*_*emp*_) < 0.05 with a one-sided test assuming TRD cases have increased CNV burden compared to healthy controls or non-TRD cases.

To test the association between known neuropsychiatric CNVs and TRD, we examined 54 CNVs nominally associated with neuropsychiatric disorders, including 12 significantly associated with schizophrenia (Supplementary Table 5) [64–66]. We included CNVs with > 50% reciprocal overlap of the region of known CNVs (--cnv-region-overlap 0.5 in PLINK), and tested if 1) carrying any of 54 neuropsychiatric CNVs was associated with TRD using logistic regression; 2) any known CNV was associated with TRD in the Fisher’s exact test (crude model) and logistic regression model (adjusted for sex and the first ten ancestral PCs). We applied Bonferroni correction for multiple comparisons (*P* < 0.05/20 = 0.0025, 20 detected in our samples).

## Results

### SNP-based heritability

Before conducting the GWAS meta-analysis, we evaluated whether our TRD definition was heritable in the largest Swedish sample of 1 487 TRD cases, 1 483 non-TRD cases, and 5 417 healthy controls. The ${h}_{{SNP}}^{2}$ of TRD risk (TRD compared to healthy controls) on the liability scale was 26% (95% confidence interval [CI] = 16–37%, *P* = 1.3 × 10^−6^) assuming a prevalence of 1.5% for severe TRD treated with ECT. For treatment resistance in MDD (TRD vs. non-TRD), the liability-scale ${h}_{{SNP}}^{2}$ was not significant at 20% (95%CI = −5–45%, *P* = 0.122, assuming a prevalence of 10% for severe TRD treated with ECT among MDD). Given the uncertainty in the population prevalence of TRD, we also reported a range of ${h}_{{SNP}}^{2}$ estimates with varying prevalence (Supplementary Fig. 1).

We observed similar results in the sensitivity analysis including 1 778 ECT-treated MDD cases, 2 264 non-ECT-treated MDD cases, and 5 417 healthy controls. The ${h}_{{SNP}}^{2}$ estimates were 29% (95%CI = 20–38%, *P* = 4.63 × 10^−10^) for ECT-treated MDD compared to healthy controls (Supplementary Fig. 1A) and 19% (95%CI = 0–38%, *P* = 0.047) for ECT-treated MDD compared to non-ECT-treated MDD (Supplementary Fig. 1B).

### GWAS meta-analysis

**Fig. 2:**
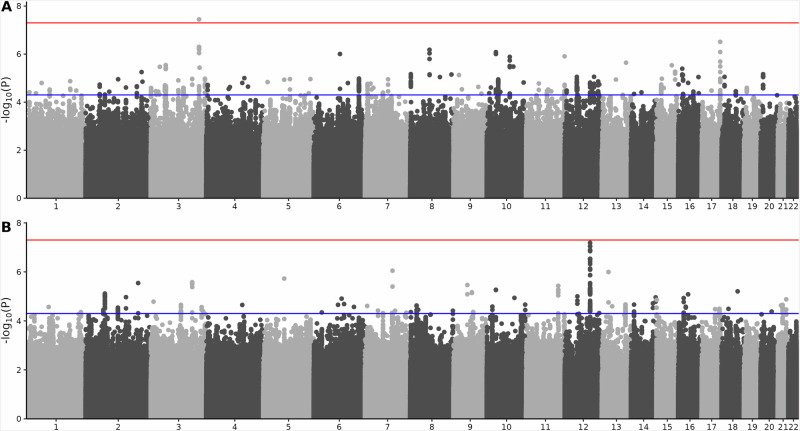
Manhattan plots of GWAS meta-analysis of TRD. **A** The Manhattan plot of GWAS meta-analysis of TRD risk. **B** The Manhattan plot of GWAS meta-analysis of treatment resistance in MDD. The red line indicates genome-wide significance at *P* < 5.0 × 10^−8^; the blue line indicates suggestive genome-wide significance at *P* < 5.0 × 10^−5^.

In total, 2 062 TRD cases, 38 544 non-TRD cases, and 441 037 healthy controls passed QC. Sample characteristics for each cohort are summarized in Table 1. We found no evidence of genomic inflation at each site ($\lambda$~1, Supplementary Table 6, Manhattan, and Q-Q plots in (Supplementary Fig. 2–7). GWAS meta-analyses of ~7 million SNPs that were present in all three samples revealed no inflation or technical bias ($\lambda$, λ_1000_ and LDSC intercept ~1, Supplementary Table 6, Manhattan plots in Fig. 2, Q-Q plots Supplementary Fig. 8). The LDSC ${h}_{{SNP}}^{2}$ estimates based on meta-analysis summary statistics were somewhat lower than the estimate from the Swedish sample, with overlapped CIs (Supplementary Fig. 9).

**Table 1:** Characteristics of samples in each study cohort.

	Sweden			EstBB			FinnGen		
	TRD	Non-TRD^a^	Healthy controls	TRD	Non-TRD	Healthy controls	TRD	Non-TRD	Healthy controls
N	1487	1483	5417	127	21,338	139,358	448	14,723	296,262
N effective	vs. healthy controls: 4667 vs. non-TRD: 2970			vs. healthy controls: 508 vs. non-TRD: 505			vs. healthy controls: 1789 vs. non-TRD: 1739		
Age at the end of follow-up, mean (SD)^b^	54.1 (16.7)	41.0 (10.4)	45.4 (7.68)	51.3 (16.2)	51.9 (16.2)	49.0 (17.1)	54.3 (16.7)	54.0 (17.9)	59.6 (17.9)
Sex, female (%)	924 (62.1)	1034 (69.7)	3002 (55.4)	99 (78.0)	16,424 (77.7)	85,537 (61.4)	285 (63.6)	9967 (67.7)	161,525 (54.5)
Ever used AD (%)	1487 (100)	581^a^ (100)	–	127 (100)	21,338 (100)	–	448 (100)	14,723 (100)	–
No. AD class, mean(Q1–Q3)^c^	2.3 (1–3)	1.4^a^ (1–2)	–	2.3 (2–3)	1.5 (1–2)	–	2.4 (2–3)	1.7 (1–2)	–
No. AD type, mean(Q1–Q3)^c^	5.0 (3–6)	1.7^a^ (1–2)	–	6.6 (5–8)	2.0 (1–3)	–	6.1 (4–8)	2.5 (1–3)	–
Ever used lithium (%)	450 (30.5)	4^a^ (0.7)	–	7 (5.5)	3 (0.01)	–	45 (10.1)	663 (4.5)	–
Ever used atypical antipsychotics (%)^d^	1085 (73.0)	13^a^ (2.2)	–	113 (89.0)	3524 (16.5)	–	400 (89.3)	3960 (26.9)	–

*AD* antidepressant, *TRD* treatment-resistant depression.

^a^In Sweden, the information is not available in one cohort (iCBT), we reported the statistics from the other two cohorts.

^b^Age at the end of follow-up: The end of follow-up is 2016-12-31 in Sweden, 2021-12-31 in EstBB and 2019-12-01 in FinnGen.

^c^AD class and AD type were defined by ATC code 5-digit for AD class and ATC code 7-digit for AD type.

^d^We included the following atypical antipsychotics: aripiprazole, olanzapine, risperidone, and quetiapine.

The GWAS meta-analysis of TRD risk identified one SNPs (rs57609176) at genome-wide significance (Fig. 2A). This SNP (MAF = 0.098 for allele A, *P* = 3.582 × 10^−8^) in the locus (3:172,591,182–172,643,970) lies in the intronic region of *SPATA16* (Supplementary Table 7, 8 and Supplementary Fig. 10A*)*. Comparing 2 409 ECT-treated MDD to healthy controls, rs57609176 remained genome-wide significant, and another SNP rs62035089 (chr15:81169259; MAF = 0.016) in low LD with nearby markers also passed genome-wide significance (Supplementary Table 7, Supplementary Table 9 and Supplementary Fig. 10B, Supplementary Fig. 11A).

For treatment resistance in MDD, we found one suggestive locus approaching genome-wide significance. The lead SNP was rs11106676 (MAF = 0.348 for allele T, *P* = 6.521 × 10^−8^), in the intergenic region of *snoU13* and *PLEKHG7* on chromosome 12 (chr12:92994875–93319627) (Supplementary Table 7, Supplementary Table 10 and Supplementary Fig. 10C). There was another suggestive locus comparing ECT-treated MDD to non-ECT-treated MDD, with lead SNP rs34867988 (MAF = 0.338 for allele A, *P* = 1.759 × 10^−7^) on chromosome 2 (chr2:60942125–61035546), upstream of *ATP1B3P1* (Supplementary Table 7, Supplementary Table 11 and 10D, Supplementary Fig. 11B).

### Shared genetics with psychiatric and cognitive traits

**Fig. 3:**
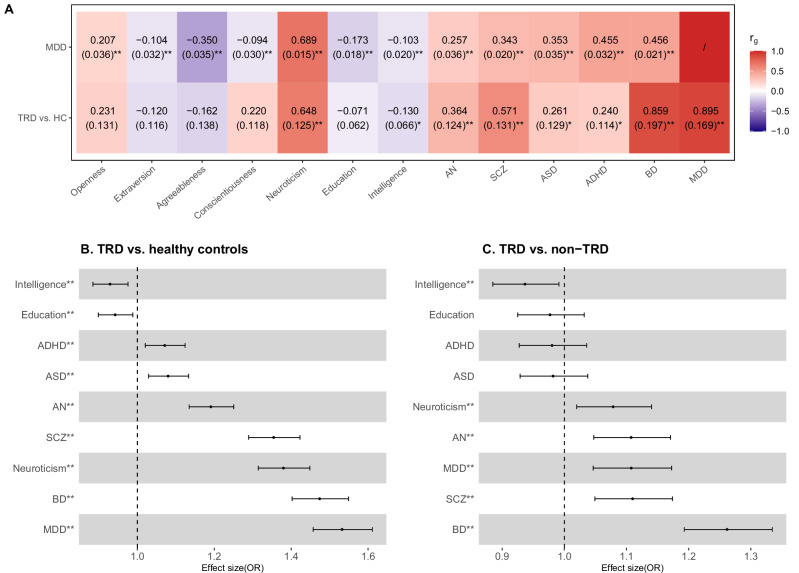
Genetic overlap of TRD with other psychiatric disorders and cognitive traits. **A** Genetic correlations (*r*_*g*_) of TRD risk with other psychiatric disorders and cognitive traits. The plot is ordered by the *r*_*g*_ of MDD and other psychiatric disorders and cognitive traits, with personality traits grouped together. ADHD attention deficit hyperactivity disorder, AN anorexia nervosa, ASD autism spectrum disorder, BD bipolar disorders, MDD major depressive disorder, SCZ schizophrenia. **B** The association between PRS of psychiatric traits and TRD risk. ORs for TRD are associated with each SD increase in the PRS of other psychiatric traits. **C** The association between PRS of psychiatric traits and treatment resistance in MDD. The traits in panels **B** and **C** are ordered by the effect sizes. * Significance at nominal *P* < 0.05; ** FDR < 0.05.

To quantify the genetic overlap between TRD and other psychiatric and cognitive traits, we first estimated *r*_*g*_ using our GWAS summary statistics of TRD and the publicly available summary statistics of other traits (Fig. 3A). We also examined the association between individual-level PRS for other traits and TRD (Fig. 3B, C).

For TRD risk (TRD vs. healthy controls), we found a high *r*_*g*_ with MDD (*r*_*g*_ = 0.90, standard error (SE) = 0.17), not significantly different from one. Notably, we observed high *r*_*g*_ between TRD risk and other severe psychiatric disorders, such as bipolar disorder (*r*_*g*_ = 0.86, SE = 0.20) and schizophrenia (*r*_*g*_ = 0.57, SE = 0.13) (Supplementary Table 12). These estimates for TRD risk were significantly different from those for MDD (*e.g*., *r*_*g*_ = 0.46 between MDD and bipolar disorder, *P* = 0.042) (Supplementary Table 12 and Supplementary Fig. 12). We also observed a positive genetic correlation of TRD risk with neuroticism (*r*_*g*_ = 0.65, SE = 0.12), whereas genetic correlation with other personality traits, including agreeableness, conscientiousness, openness, and extraversion, did not reach statistical significance. We observed similar estimates for the ECT-treated MDD compared to healthy controls (Supplementary Fig. 12, Supplementary Fig. 13A).

For treatment resistance in MDD (TRD vs. non-TRD), genetic correlation estimates had large standard errors and were non-significant, indicating limited statistical power (Supplementary Fig. 13B and Supplementary Table 12). We, therefore, tested the associations between PRS of the nine selected traits with TRD risk and treatment resistance in MDD.

The analysis with PRS revealed positive associations of TRD risk with PRS of all included psychiatric disorders, and negative associations with educational attainment and intelligence. Except for educational attainment, all associations were significant at FDR < 0.05 (Fig. 3B and Supplementary Table 13). TRD risk was most strongly associated with MDD PRS (OR = 1.53 per SD increase in the PRS, 95%CI = 1.46–1.61), bipolar disorder (OR = 1.47, 95%CI = 1.40–1.55), and schizophrenia (OR = 1.35, 95%CI = 1.29–1.42).

Similarly, treatment resistance in MDD showed significant positive associations with PRS for bipolar disorder, schizophrenia, MDD, anorexia nervosa, and neuroticism. Interestingly, treatment resistance in MDD had a weaker association with MDD PRS (OR = 1.11, 95%CI = 1.05–1.17) compared to bipolar disorder PRS (OR = 1.26, 95%CI = 1.19–1.33). We found treatment resistance in MDD was negatively associated with PRS of intelligence (OR = 0.94, 95%CI = 0.88–0.99) (Fig. 3C and Supplementary Table 13). We observed similar results in ECT-treated MDD compared to healthy controls and non-ECT-treated MDD (Supplementary Fig. 14). A sensitivity analysis further excluding psychotic MDD cases (leaving 1 102 TRD cases in the analysis) showed similar results (Supplementary Fig. 15, 16).

### CNV global burden

In total, 21 057 large CNVs were called by at least two CNV methods and passed QC in Swedish samples. In the analysis, 10 786 large (>100 kb) and rare (frequency < 1%) CNVs, including 4 211 deletions and 6 575 duplications in 9 457 individuals, were included. Compared to healthy controls or non-TRD, TRD cases had a significantly higher burden of deletions regarding the total length and the average length. Per 100 kb increase in the length of deletions was associated with TRD risk (OR = 1.02–1.03, *P*_*emp*_ = 0.002–0.006) and treatment resistance in MDD (OR = 1.03–1.04, *P*_*emp*_ = 0.009–0.031). TRD appeared to have more deletions than healthy controls or non-TRD cases, but the associations were not significant. We did not find evidence supporting the enrichment of duplications in TRD (Table 2). The results for ECT-treated MDD showed similar levels of associations (Supplementary Table 14).

**Table 2:** Global burden test of deletion/duplication CNVs in TRD.

		Deletions		Duplications	
		OR	*P*_*emp*_	OR	*P*_*emp*_
TRD vs. healthy controls	Number of CNV	1.06	0.122	0.88	1.000
	Total length^*^	1.02	0.006	0.97	1.000
	Average length^*^	1.03	0.002	0.97	1.000
TRD vs. non-TRD	Number of CNV	1.08	0.115	0.98	0.657
	Total length^*^	1.03	0.031	0.99	0.721
	Average length^*^	1.04	0.009	1.00	0.483

^*^The total and average length of CNV is in the unit of 100 Kb.

### Known neuropsychiatric CNV and TRD

We further examined if previously reported neuropsychiatric CNVs were associated with TRD. Our sample included 20 of 54 neuropsychiatric CNVs, detected in 395 individuals (frequency < 1% per CNV). Carrying any of these CNVs was associated with significantly higher odds of TRD compared to healthy controls (OR = 1.74, 95%CI = 1.18–2.58) and non-TRD (OR = 2.86, 95%CI = 1.54–5.32). ECT-treated MDD was also associated with these CNVs compared to healthy controls (OR = 1.61, 95%CI = 1.10–2.36) and non-ECT-treated MDD (OR = 1.58, 95%CI = 1.00–2.58).

**Fig. 4:**
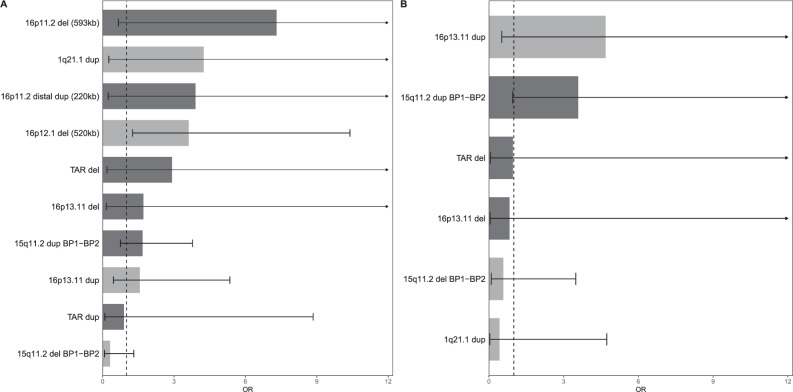
Associations of known neuropsychiatric CNVs and TRD or treatment resistance in MDD. The figure only showed those with CNV detected by both TRD and the comparison groups, adjusting for sex and the first ten ancestral PCs. The analysis was conducted in the Swedish cohort. **A** The association between neuropsychiatric CNV and TRD risk when comparing TRD vs healthy controls. **B** The association between neuropsychiatric CNV and treatment resistance in MDD when comparing TRD vs non-TRD. In both panels, the CNVs were ordered by the ORs.

Examining these individual CNVs, 16p12.1 del (520 kb) was nominally associated with a higher risk of TRD (OR = 3.62, 95%CI = 1.26–10.40, *P* = 0.017) and ECT-treated MDD (OR = 3.02, 95%CI = 1.05–8.67, *P* = 0.040) compared to healthy controls (Fig. 4, Supplementary Table 15, Supplementary Fig. 17). No non-TRD cases carried this CNV. A few other known CNVs, including 16p11.2 del (593 kb), 1q21.1dup, and 16p11.2 distal dup (220 kb), were more common in TRD cases than in controls but did not show significant associations.

## Discussion

Leveraging the register records of patient diagnosis, medical procedures, and prescription drugs from three Nordic countries, our study focused on severe TRD cases treated with ECT and antidepressants and investigated common variants and rare CNVs in TRD cases compared to healthy controls and non-TRD. We found a significant ${h}_{{SNP}}^{2}$ of 26% for TRD risk and 20% for treatment resistance in MDD (non-significant) in the Swedish sample. The subsequent GWAS meta-analysis with ~ 84 000 MDD cases (including 2 062 severe TRD cases) revealed a genome-wide significant locus for TRD risk and a suggestive locus for treatment resistance in MDD. TRD showed high genetic correlations with MDD (*r*_*g*_ = 0.90), bipolar disorder (*r*_*g*_ = 0.86), and schizophrenia (*r*_*g*_ = 0.57). Further analyses using PRS indicated that TRD had higher common-variant burdens of psychiatric disorders compared to non-TRD cases. TRD risk and treatment resistance in MDD were negatively associated with the PRS for cognitive traits. Additionally, TRD cases carried larger rare CNVs compared to healthy controls and non-TRD. Known neuropsychiatric CNVs were associated with elevated TRD risk, with the 16p12.1 deletion (520 kb) nominally associated with ~ 3.6 times higher TRD risk compared to healthy controls.

Our study added evidence supporting the heritable component in TRD attributable to common variants. The ${h}_{{SNP}}^{2}$ of TRD risk is comparable with previous studies [13–15]. For treatment resistance in MDD, the ${h}_{{SNP}}^{2}$ estimates in the literature varied widely with different definitions of TRD. Earlier studies using self-reported questionnaires for antidepressant response, generally reported unreliable estimates [14, 15], whereas clinical trials or EHR studies reported significant estimates of around 13% [13, 67]. Our estimate of 20% for treatment resistance in MDD was similar but had a wide CI because of the relatively small numbers of ECT-treated individuals even in large biobanks. New avenues are emerging to enhance statistical power in the ECT-based TRD definition using EHR data [16].

We identified one genome-wide significant locus, and one suggestive locus associated with TRD. The significant locus on chromosome 3, associated with TRD risk, was within the intronic region of *SPATA16*. *SPATA16* is known for its role in spermatogenesis and is associated with globozoospermia, MDD, brain measurements, body mass index, and insomnia [68–72]. The suggestive locus on chromosome 12, involving genes *snoU13* and *PLEKHG7*, was associated with treatment resistance in MDD but has not been implicated for psychiatric traits, warranting further investigation. When comparing ECT-treated MDD to non-ECT-treated MDD, the lead SNP (rs34867988) on chromosome 2 was in high LD (r^2^ = 0.93) with the SNP rs62149375, which is linked to general cognitive ability and educational attainment [73, 74]. All three loci were nominally significant in a recent GWAS meta-analysis on MDD [71, 75], and a TRD study based on predicted ECT probability [16], strengthening their relevance (Supplementary Table 16). However, previously reported TRD loci did not replicate in our study, possibly due to differences in TRD phenotypes and sample ascertainment (Supplementary Table 17). While our study yielded promising results, limited statistical power hindered further functional annotation and pathway analysis. Future investigation is essential to elucidate the functional significance of these genetic associations in TRD susceptibility.

Our results highlight TRD as a subtype of MDD with a partially distinct genetic profile. While TRD and MDD had a high *r*_*g*_, TRD showed notably higher *r*_*g*_ with severe psychiatric disorders such as schizophrenia and bipolar disorder, being twice higher than those for MDD. Similar as reported previously [13, 16, 31, 67], we found strong associations between these psychiatric disorders PRS and TRD, even when excluding psychotic MDD (Supplementary Fig. 15, 16). However, the high genetic correlation of TRD with schizophrenia and bipolar disorder may be specific to our severe TRD cases. A previous study showed moderate genetic correlations between TRD and schizophrenia and bipolar disorder (*r*_*g*_ = 0.21–0.35), likely due to its broader definition of TRD (*i.e*., individuals with MDD diagnosed from primary care and with at least two antidepressant switches) [13]. Given that TRD is generally considered as a more severe form of MDD than other MDD cases, especially when defined by ECT, our results suggest a closer genetic relationship between severe TRD cases and other severe psychiatric disorders.

TRD showed genetic associations with cognitive impairment. The suggestive locus on chromosome 2 in our GWAS of ECT-treated MDD versus non-ECT-treated MDD has previously been associated with cognitive traits [73, 74]. Negative genetic correlations and PRS associations with cognitive traits align with previous research on MDD, antidepressant response, and TRD [31, 67]. Similarly, treatment-resistant schizophrenia has shown a negative genetic correlation with education attainment and cognitive ability [76]. Taken together, these findings suggest a potential genetic relationship between impaired cognitive traits and treatment resistance in psychiatric disorders. However, conflicting evidence from another study reported a positive genetic overlap between TRD and cognitive traits [16], indicating the need for further in-depth investigations.

To the best of our knowledge, this study is the first to investigate rare CNVs in severe TRD patients who received ECT. Our findings suggest an important role of rare CNV in TRD and underscore the need for comprehensive genetic studies to unravel the complex underpinnings of TRD. First, we observed a significant enrichment of rare and large CNV burden in TRD compared to non-TRD/controls, reinforcing the heritable component of TRD. Second, we found an association between a group of neuropsychiatric CNVs and TRD (OR = 1.74–2.86 depending on the phenotype), which appears stronger than the association with MDD (OR = 1.34) [66], indicating a stronger link between neuropsychiatric CNVs and TRD than with MDD. Moreover, the 16p12.1 deletion (520 kb) showed a nominal association with TRD, with effects comparable to its association with schizophrenia (OR = 3.3) [65]. This CNV has been linked to learning disability and congenital anomalies [66, 77]. Future studies with improved statistical power are needed to replicate our findings and explore genome-wide CNVs.

Leveraging register records and biobanks from three Nordic countries, our study investigated common variants and rare CNV burdens in TRD treated by ECT, providing valuable insights into the genetic architecture of TRD. We focused on the subset of ECT-treated cases [16, 31], who were likely to have received ECT due to treatment resistance. TRD is highly polygenic, with common genetic variants contributing small additive effects. Rare variants and structural variations may have stronger effects but require robust statistical power for detection. Our study provides a foundational framework for future research on TRD genetics. Consortium efforts (*e.g*., the GenECT consortium) gathering more samples with severe phenotypes are warranted to replicate our findings and enable further exploration [78]. High-throughput technologies, such as exome and whole-genome sequencing, offer opportunities to gain deeper insight into actionable variants and functional pathways [24].

Despite our efforts, our analysis is limited by statistical power, especially regarding treatment resistance in MDD. The lack of primary care data in some cohorts means we captured a subset of severe MDD cases. However, TRD is often considered a more severe form of MDD than other MDD cases, and we focused on cases who received ECT, which is typically administered in specialist care. We further required at least one adequate antidepressant trial before ECT as a proxy for the indication of treatment resistance. Indeed, cases meeting our TRD criteria had significantly higher use of antidepressants and augmentation therapies (lithium and atypical antipsychotics) compared to other cases (Table 1). In addition, our prior study demonstrated consistent genetic findings across ECT-based definitions with varying stringency, suggesting similar underlying genetic components [30]. Future studies comparing different TRD definitions are warranted. Nevertheless, future research with data from both primary and specialist care is crucial to validate our findings. Moreover, due to the limited number of non-European ancestry samples, our analyses focused on European ancestry; further studies with diverse ancestry are needed.

In conclusion, our findings add evidence of heritable components of TRD risk and treatment resistance in MDD and yield valuable insights into the genetic underpinnings of TRD and its genetic overlap with psychiatric disorders and cognitive traits. Our work highlights the importance of investigating rare CNVs in TRD. These discoveries set the stage for future research in this ‘difficult-to-treat’ condition.

## Supplementary information

Supplementary materials

Supplementary tables

## Data Availability

The individual data cannot be shared publicly due to ethical and consent restrictions. Summary statistics can be shared upon request.

## Code availability

Codes and scripts used for phenotype derivation and statistical analysis can be shared upon request.
