# Supplementary material for: Genome-wide association meta-analysis and rare copy number variant analysis of treatment-resistant depression
Source: Mol Psychiatry. 2025 Jun 26;30(11):5024–33. doi: 10.1038/s41380-025-03084-z (PMC12532596; doi:10.1038/s41380-025-03084-z)
Supplement: Supplementary file 1 — Supplementary materials [file 41380_2025_3084_MOESM1_ESM.pdf]

Supplementary materials for:

Genome-wide association meta-analysis and rare copy number variant analysis of treatment-resistant depression

Table of Contents

Supplementary Methods 2

Supplementary Figure 1. Range figure of h^2_{SNP} on the liability scale. 8

Supplementary Figure 2. Manhattan plot and Q-Q plot for GWAS on ECT-treated MDD or TRD risk (ECT-treated MDD or TRD vs. healthy controls) in Sweden. 9

Supplementary Figure 3. Manhattan plot and Q-Q plot for GWAS on ECT-treated MDD or TRD risk (ECT-treated MDD or TRD vs. healthy controls) in Estonia Biobank. 10

Supplementary Figure 4. Manhattan plot and Q-Q plot for GWAS on ECT-treated MDD or TRD risk (ECT-treated MDD or TRD vs. healthy controls) in FinnGen. 11

Supplementary Figure 5. Manhattan plot and Q-Q plot for GWAS on treatment resistance in MDD (TRD vs. non-TRD and ECT-treated MDD vs. non-ECT-treated MDD) in Sweden 12

Supplementary Figure 6. Manhattan plot and Q-Q plot for GWAS on treatment resistance in MDD (TRD vs. non-TRD and ECT-treated MDD vs. non-ECT-treated MDD) in Estonia Biobank. 13

Supplementary Figure 7. Manhattan plot and Q-Q plot for GWAS on treatment resistance in MDD (TRD vs. non-TRD and ECT-treated MDD vs. non-ECT-treated MDD) in FinnGen. 14

Supplementary Figure 8. Q-Q plot for GWAS meta-analysis on ECT-treated MDD/TRD risk (TRD vs. healthy controls) and treatment resistance in MDD (ECT-treated MDD vs. non-ECT-treated MDD and TRD vs. non-TRD). 15

Supplementary Figure 9. Estimates of h^2_{SNP} on the liability scale using LDSC. 16

Supplementary Figure 10. Regional plots for top loci 17

Supplementary Figure 10 (con't). Regional plots for top loci 18

Supplementary Figure 11. Manhattan plots of GWAS meta-analysis 19

Supplementary Figure 12. Forest plot of genetic correlation between ECT-treated MDD or TRD risk and other psychiatric or cognitive traits. 20

Supplementary Figure 13. Genetic correlations (r_g) of ECT 21

Supplementary Figure 14. Associations between PRS of other psychiatric disorders and cognitive traits and ECT 22

Supplementary Figure 15. The association between PRS of psychiatric traits and ECT-treated MDD/TRD risk excluding psychotic MDD in Swedish samples (case-control comparison) 23

Supplementary Figure 16. The association between PRS of psychiatric traits and treatment resistance in MDD excluding psychotic MDD in Swedish samples (case-case comparison). 24

Supplementary Figure 17. Associations of known neuropsychiatric CNVs and ECT-treated MDD. 25

Supplementary Methods

Methods S1. Descriptions of studies in GWAS meta-analysis

Sweden

We used the combined samples from three Swedish cohorts to conduct analysis in Sweden: 1) the Predictors for ECT (PREFECT) study, a Swedish study enrolled electroconvulsive therapy (ECT)-treated patients from the Swedish national quality register (2013-2017)[1]. PREFECT participants were included in this study as TRD cases if they were treated with ECT in the context of major depressive disorder (MDD); 2) The internet-based cognitive behavior therapy (iCBT) study, which enrolled adult MDD patients who received internet-based cognitive behavior therapy at the Internet Psychiatry Clinic in Stockholm[2]; 3) Swedish Twin Studies of adults: Genes and Environment (STAGE), from a part of Swedish twin registries and including middle-aged twins as study participants[3]. Detailed information about samples was described previously[1–3]. The study was approved by the Swedish Ethical Review Authority (Dnr: 2012/1969-31/1, 2009/1089-31/2, 2023-03073-01).

We adopted the same definitions used in the previous study[4]. According to the TRD definition, TRD cases are derived only from the PREFECT study, and non-TRD cases are derived from the iCBT and STAGE study. In addition, we extracted healthy controls from the STAGE study. In the PREFECT study, we linked the prescribed drug registry (PDR) to obtain medication data and then defined the antidepressant treatment duration for deriving TRD cases. Since the iCBT cases were mild-moderate MDD cases that began treatment with internet-based cognitive behavior therapy, they were unlikely to be treated with ECT. Therefore, we considered these MDD cases as non-TRD. In STAGE samples, using linked medication data from the PDR, we excluded individuals with ECT treatment and diagnosis of schizophrenia (SCZ), and bipolar disorder (BD) based on diagnosis and operation data from the linked national patient registry. Moreover, we defined antidepressant treatment duration and then included those MDD cases with antidepressant use but no more than two antidepressants with adequate duration. The healthy controls were individuals without a diagnosis of MDD, SCZ, and BD.

Genotyping for all 3 studies used Illumina Infinium Global Screening Arrays (v1). PREFECT and iCBT samples were genotyped in Life and Brain GmbH (Bonn, Germany). STAGE samples were genotyped by the SNP&SEQ Technology Platform (Uppsala, Sweden).

We first excluded SNPs of monomorphic sites, strand-ambiguous, low minor allele frequency (>0.01), and then we harmonized the markers and allele coding to merge raw genotype data. In the merged data, 12 666 individuals and 499 317 SNPs were included. We ran quality control (QC) using the PGC Ricopili pipeline[5]. Firstly, we removed SNPs with missingness >5%, and 69 samples with the following criteria: 1) sample call rate <0.98; 2) excessive heterozygosity (FHET outside +/- 0.2): 3) sex mismatch. Then we excluded SNPs due to 1) call rate per SNP <0.98; 2) invariant; 3) Hardy-Weinberg disequilibrium ($P<1\times10^{-6}$ in controls and cases separately); 4) difference in call rate before cases and controls >0.01; 5) MAF <0.01. We kept 459 906 SNPs after QC. Subsequently, we conducted principal components analysis (PCA) with reference panels of 1000 Genomes of the global population and plotted the first two principal components (PCs) to check the ancestral outliers (the first two PCs exceeded 6 standard deviations from the mean value of the European samples in the reference population). We identified and excluded 173 non-European ancestral outliers. Furthermore, relatedness was estimated from genotype data, and the proportion of the genome shared identical-by-descent ($\hat{\pi}$) was estimated. We removed 1 318 individuals from pairs of related samples ($\hat{\pi}>0.2$). In total, 11 106 individuals were retained after QC. Using

post-QC genotyped data, we went through imputation with a reference panel of Haplotype reference Consortium data (HRC 1.1) in the Sanger imputation service. EAGLE2+IMPUTE2 and PBWT were used for pre-phasing and imputation[6–9]. The genome build was hg19.

GWAS for TRD in Sweden was performed with PLINK2 on hard-call imputed genotype data, with sex and the first five principal components as covariates.

Estonia Biobank

Estonian Biobank (EstBB) is a population-based cohort with a rich variety of phenotypic and health-related information collected for each participant[10, 11]. At recruitment, participants signed a consent allowing follow-up linkage of their electronic health records (EHR), thereby providing a longitudinal collection of their phenotypic information. The EstBB database includes health records from the National Health Insurance Fund Treatment Bills (from 2004), Tartu University Hospital (from 2008), and North Estonia Medical Center (from 2005), and data from different registries (causes of death, cancer, etc.). Disease diagnoses are recorded based on the International Classification of Diseases, 10th revision (ICD-10 codes) and prescribed medication according to the Anatomical Therapeutic Chemical (ATC) classification system. Information on clinical procedures is also recorded with health insurance fund treatment service codes. The activities of the EstBB are regulated by the Human Genes Research Act, which was adopted in 2000 specifically for the operations of the EstBB. Individual-level data analysis in the EstBB was carried out under ethical approval number 1.1-12/624 from the Estonian Committee on Bioethics and Human Research (Estonian Ministry of Social Affairs).

All EstBB participants were genotyped using Illumina global screening arrays (GSAv1.0, GSAv2.0, and GSAv2.0_EST) at the Core Genotyping Lab of the Institute of Genomics, University of Tartu, with quality control conducted according to best practices. During the quality control, all individuals with a call rate <95% or mismatching sex defined based on the heterozygosity of the X chromosome and sex in the phenotype data were excluded from the analysis. Variants were filtered by call rate <95% and HWE p-value $<1\times10^{-4}$ (autosomal variants only). Variant positions were updated to Genome Reference Consortium Human Genome Build 37, and all variants were changed to be from the TOP strand using GSAMD-24v1-0_20011747_A1-b37.strand.RefAlt.zip files from https://www.well.ox.ac.uk/~wrayner/strand/webpage. Prephasing was done using the Eagle v2.3 software[7] (the number of conditioning haplotypes Eagle2 uses when phasing each sample was set to: --Kpbwt=20000) and imputation was carried out using Beagle v.18May20.d20[12, 13] with an effective population size ne=20,000. As a reference, an Estonian population-specific imputation reference of 2297 WGS samples was used[14]. Further, EstBB samples were combined with the 1000 genomes phase 3 dataset for ancestry analysis. Genetic principal components were calculated using a subset of quality-controlled and pruned genotyped SNPs. This was further used to identify and remove samples that deviated from the main cluster.

TRD phenotypes in EstBB were defined according to the phenotype section of the paper. To identify individuals with ECT, we used Health Insurance Fund treatment service code 7609 (in Estonian “Elekterkrampravi”). The individuals with MDD were selected based on ICD-10 codes F32.x – F33.x. Digital drug dispensing data for antidepressants (N06Axxx ATC codes) from the Estonian National Health Insurance Fund was used to define adequate antidepressant use. The data consists of information regarding the date of drug prescription and purchase, which was analyzed to calculate adequate AD use. Individuals with BD or SCZ diagnoses (ICD-10 codes F20.x, F23.1, F23.2, F25.x, F30.x, F31.x) were excluded from the entire analysis. GWAS for TRD was performed with REGENIE 3.0.3[15], adjusting for the ten PCs, sex, and birth year, which also accounts for case-control imbalance. Data analysis was carried out in part in the High-Performance Computing Center of the University of Tartu.

PRS analysis was conducted according to the PRS methods described in the methods section in the main text. For each individual, we calculated PRSs based on the weighted GWAS summary statistics using PLINK (version 2.0). To perform a logistic regression between treatment resistance and PRSs of psychiatric-related traits, we first excluded relatives. One member per related individual pairs (PLINK PI_HAT > 0.2) by prioritising cases was removed from the association testing where PRSs were considered. This was done using an in-house script. PRSs were standardised using a *scale* function, and logistic regression was conducted using R software.

FinnGen

We conducted an analysis in FinnGen. FinnGen consists of prospectively recruited samples and a series of legacy cohorts with genotypes already available, linked with Finnish health registries for digital health records (~356 000 samples with combined genotype and health registry data)[16]. Participants in FinnGen provided informed consent for biobank research on the basis of the Finnish Biobank Act. Alternatively, separate research cohorts, collected before the Finnish Biobank Act came into effect (in September 2013) and the start of FinnGen (August 2017) were collected on the basis of study-specific consent and later transferred to the Finnish biobanks after approval by Fimea, the National Supervisory Authority for Welfare and Health. Recruitment protocols followed the biobank protocols approved by Fimea. The Coordinating Ethics Committee of the Hospital District of Helsinki and Uusimaa (HUS) approved the FinnGen study protocol (number HUS/990/2017).

The FinnGen study is approved by the Finnish Institute for Health and Welfare (THL) (approval number THL/2031/6.02.00/2017, amendments THL/1101/5.05.00/2017, THL/341/6.02.00/2018, THL/2222/6.02.00/2018, THL/283/6.02.00/2019, THL/1721/5.05.00/2019 and THL/1524/5.05.00/2020), the Digital and Population Data Service Agency (VRK43431/2017-3, VRK/6909/2018-3 and VRK/4415/2019-3), the Social Insurance Institution (KELA) (KELA 58/522/2017, KELA 131/522/2018, KELA 70/522/2019, KELA 98/522/2019, KELA 134/522/2019, KELA 138/522/2019, KELA 2/522/2020 and KELA 16/522/2020), Findata (permit numbers THL/2364/14.02/2020, THL/4055/14.06.00/2020, THL/3433/14.06.00/2020, THL/4432/14.06/2020, THL/5189/14.06/2020, THL/6619/14.06.00/2020, THL/209/14.06.00/2021, THL/688/14.06.00/2021, THL/1284/14.06.00/2021, THL/1965/14.06.00/2021, THL/5546/14.02.00/2020, THL/2658/14.06.00/2021, THL/4235/14.06.00/2021) and Statistics Finland (permit numbers: TK-53-1041-17 and TK/143/07.03.00/2020 (earlier TK-53-90-20) TK/1735/07.03.00/2021).

Prospective samples were genotyped using the ThermoFisher Axiom custom array which tags a total of 655,973 variants. Genotype calling was performed using the Array Power Tools software. Legacy cohorts were genotyped using various Illumina arrays and genotype calling was performed using either GenCall or zCall algorithms.

For both prospective and legacy cohorts the following quality control metrics were used.

Samples were removed if:

- Pihat was >0.9 and the samples were not monozygotic or replicates
- There was a discrepancy between reported sex and genetically determined sex (F-value ≤0.3 for females and ≥0.8 for males)
- Missingness was ≥5%
- Heterozygosity was ±4 standard deviations from the population average
- Pihat was >0.1 with 14 or more samples
- Samples were ±4 standard deviations away from the population average according to the first two genetic principal components.

Samples were tagged should there be evidence of a mendelian error or contain replicate samples with over 50,000 discrepancies.

Variants were removed if:

- The variant failed the Hardy-Weinberg Equilibrium test (p-value < 10^{-6})
- The variant had a call rate <98%

Pre-phasing was performed using Eagle 2.3.5[7] and samples were imputed using the SiSu v3 imputation reference panel. This reference panel is specific to the Finnish population, containing high-coverage (25-30x) whole-genome sequencing data from 3,775 Finns and 16,962,023 variants with minor allele count ≥ 3. After imputation, 16,387,711 variants were imputed with high quality (INFO >0.6).

To extract info on genetic ancestry, firstly, the FinnGen samples were combined with the 1000 genomes phase 3 dataset[17]. Genetic principal components were calculated using a subset of 49,451 pruned SNPs. Aberrant[18] was used to identify and remove samples that deviated from the main cluster. A probability of belonging to either a North-Western European or Finnish population was calculated by firstly performing PCA with individuals belonging to these ancestries from 1000 genomes data. FinnGen samples were then projected onto this PCA space and Mahalanobis distances calculated for each sample against each of the two ancestries. Samples were retained if there was ≥95% probability of belonging to the Finnish ancestry cluster.

GWAS was conducted in SAIGE[19], adjusted for 10PCs, imputed sex, age at death/end of follow-up, chips, and batches, controlling for case-control imbalance.

In addition, the descriptions of cohorts are summarized in ***Supplementary Table 1***. Before meta-analysis, we estimate r_g across three study sites. Due to limited TRD case numbers in FinnGen and Estonia Biobank, we did not obtain an estimate for r_g between them. The range of r_g is broad (10%-59%), with large standard error, thus, the estimate is not precise. To ensure the quality of the meta-analysis, we did further check after the meta-analysis on test statistic inflation and LDSC intercept (***Supplementary Table 6***).

Methods S2. Phenotype definitions

TRD. We derived TRD definitions by using information on ECT treatment and antidepressant use, which has been proposed and validated previously[4]. ECT is second- or third-line therapy for treatment failure of antidepressants[20, 21]. To avoid including ECT-treated cases due to psychotic symptoms or life-threatening conditions[22], we further checked the medication data on antidepressants before the first ECT treatment to ensure that MDD cases received ECT due to treatment resistance. We derived the treatment duration for each antidepressant based on the first and last dispense date of the antidepressant and considered adequate treatment duration as ≥ 6 weeks to account for the length of therapeutic effect and distinguish it from adverse effects[23, 24]. The different treatment episode was defined as the gap time between two consecutive dispenses of more than 120 days[25]. The exclusion criteria for TRD cases were those with a diagnosis of SCZ or BD.

Non-TRD. We defined the non-TRD as the individuals with MDD diagnosis, antidepressant use but no more than two antidepressant treatments with adequate duration, and no ECT treatment during the study period. We also excluded individuals with a diagnosis of SCZ or BD.

ECT-treated MDD. To make it comparable with other TRD definitions utilized in previous research[26], we provided an alternative definition, referred to as ECT-treated MDD, if the patients meet both criteria below: 1) having at least one MDD diagnosis and 2) receiving ECT treatment.

Non-ECT-treated MDD. We defined non-ECT-treated MDD as individuals with MDD diagnosis and without ECT treatment.

Healthy controls. Healthy controls are individuals without a diagnosis of SCZ, BD, or MDD.

Phenotype derivation and related codes for disease diagnosis, medication, and ECT treatment are shown in ***Supplementary Table 2***.

Methods S3. CNV calling and analysis

CNV calling

Firstly, we derived CNVs for each individual using the above three methods separately. Then we conducted quality control for CNVs called by each method (details in Table S3). We annealed adjoining CNVs if the called region was ≥80% of the entire region to merge CNVs artificially split by the CNV calling algorithm. The basic QC criteria were to exclude CNVs with 1) low-confidence CNV and CNV region spanned across ≤10 probes; 2) CNV size ≤100 kb; 3) >50% reciprocal overlap with large genomic gaps, rearrangement segments, or segmental duplications. Next, we intersected CNVs detected by any two of three CNV calling algorithms by applying the filter on >50% reciprocal overlap for the same CNV type in the same sample. Individuals with excessive CNV calls scattered across chromosomes (>30 CNVs) were excluded. We further excluded common CNVs (frequency ≥1%) as common CNVs are generally well-tagged by SNPs[27].

CNV analysis – covariates inclusion

We followed the analysis reported in the previous study to include covariates[28]. To avoid spurious associations, we tested a list of potential confounders, including sex and the first 10 ancestry PCs. For each CNV feature, we fitted linear regression models to test the association between CNV features (dependent variable) and each potential confounder (sex and the first 10 PC components for population stratification, as predictors in the model). Then we applied the FDR method for multiple test correction. If any of the predictors remain significant (FDR <0.05), we included the predictor in the association test between CNV and TRD.

Methods S4. Genomic annotation in FUMA

We used FUMA to annotate the loci identified in the GWAS meta-analysis[29]. For GWAS of TRD risk (TRD compared to healthy controls), we only characterized genomic loci for those reaching genome-wide significance ($P<5.0\times10^{-8}$). The first r^2 threshold to define independent significant SNPs was ≥0.6 and the second r^2 threshold to define lead SNPs was ≥0.1. The LD blocks window was 250kb, and the reference panel population was 1000G Phase3 European population. For MDD treatment resistance (TRD compared to non-TRD), due to limited sample size, we did not identify any genome-wide significant SNPs. However, we observed two suggestive peaks from Manhattan plots. Thus, we lowered the maximum P-value of lead SNPs from 5.0×10^{-8} to 5.0×10^{-6} to annotate those two suggestive loci. To illustrate the locus, we made the region plot of each locus by LocusZoom[30].

Methods S5. Generating PRS

Before generating PRS, we performed additional QC on individual genetic data by excluding SNPs with 1) MAF<0.1 or INFO score<0.9; 2) duplicated SNPs; 3) strand-ambiguous SNPs; and 4) SNPs in the major histocompatibility complex region (chr6:28-34Mb). We used SbayesR to rescale the summary statistics to account for linkage disequilibrium (LD)[31].

SbayesR was chosen because of its superior performance over other methods in psychiatric disorders[32]. The summary statistics were from the same source of analysis in LDSC and to deal with potential sample overlap between discovery GWAS and target samples, either leave-one-out summary statistics were used for generating PRSs or overlapping individuals were excluded from analyses (***Supplementary Table 4***). Then, we calculated PRS for each individual summing the number of risk alleles weighted by effect size per standard error (SE) across the whole genome in PLINK2.0[33].

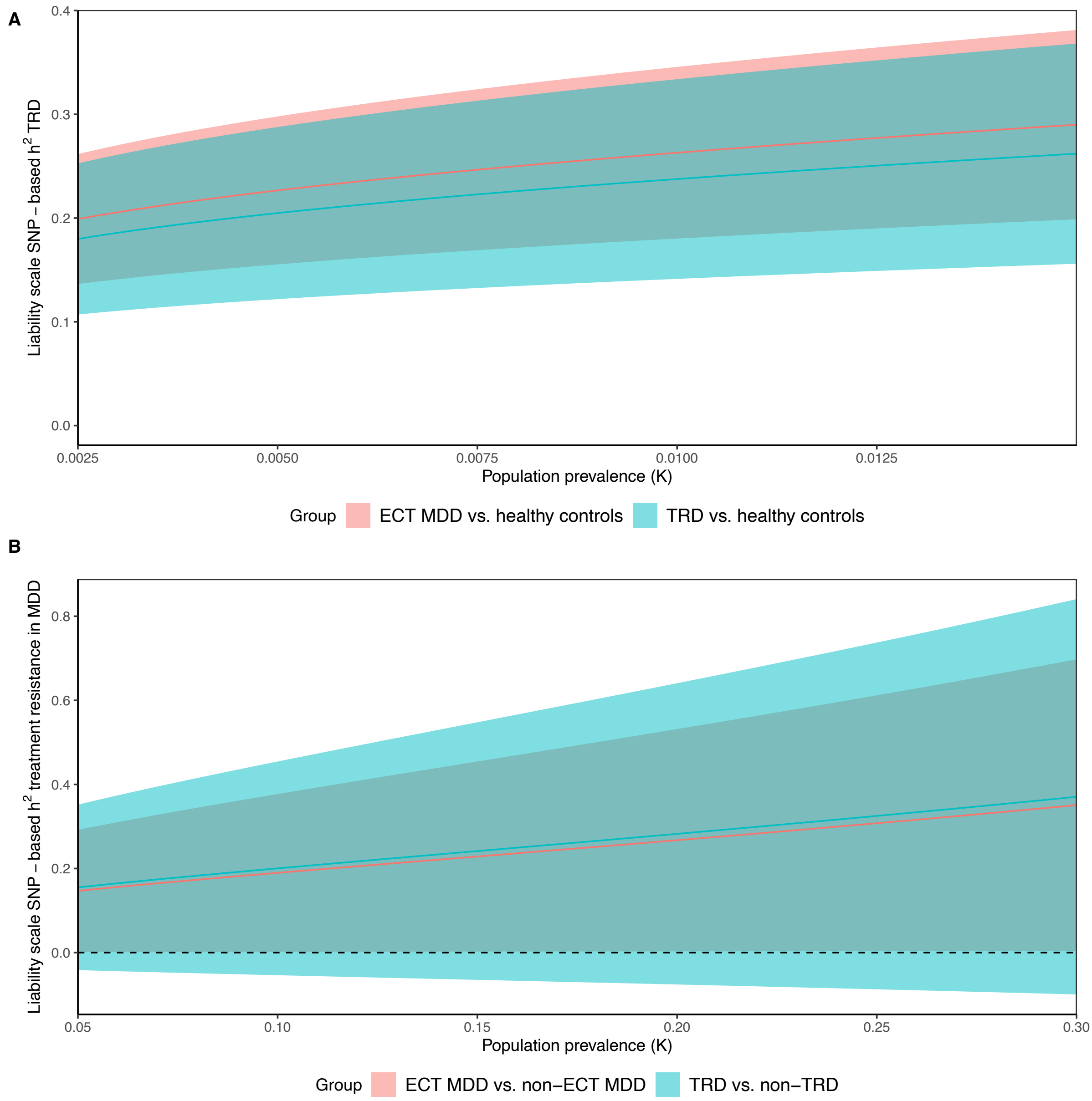

Supplementary Figure 1. Range figure of h^2_{SNP} on the liability scale. The plot of h^2_{SNP} on the liability scale by assuming a range of prevalence of TRD, non-TRD and healthy controls. The h^2_{SNP} was estimated in the Swedish cohort.

A) Liability scale of h^2_{SNP} of TRD risk. Pink colour: ECT-treated MDD vs healthy controls. Turquoise colour: TRD vs healthy controls

B) Liability scale of h^2_{SNP} of treatment resistance in MDD. Pink colour: ECT-treated MDD vs non-ECT-treated MDD. Turquoise colour: TRD vs non-TRD

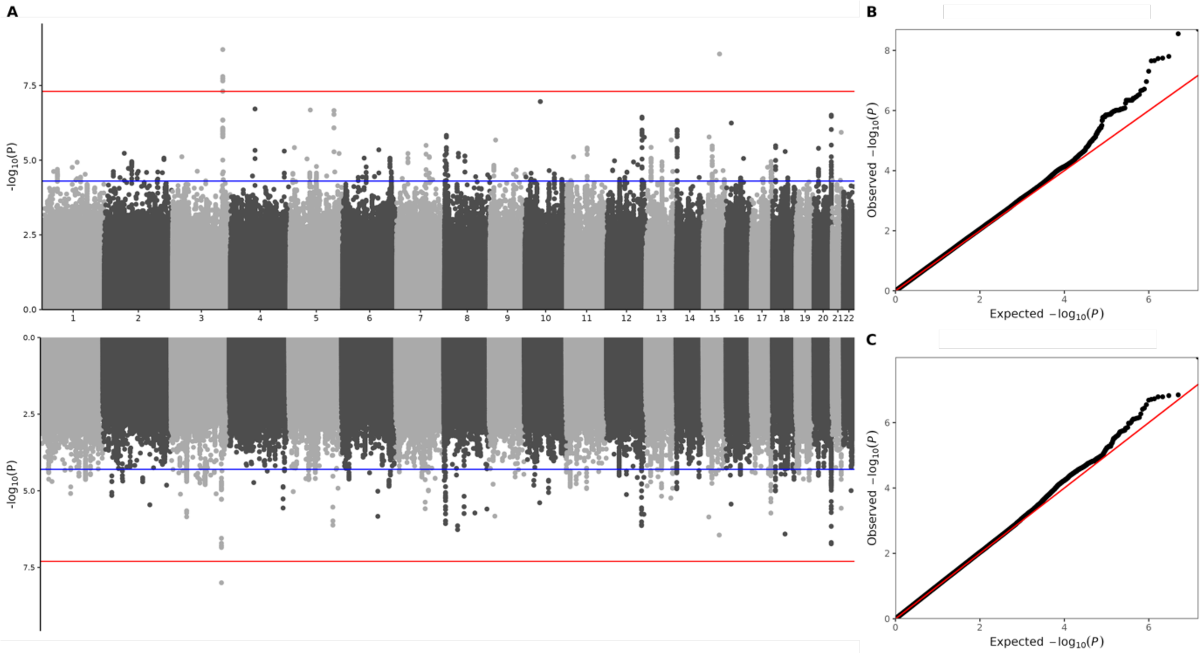

Supplementary Figure 2. Manhattan plot and Q-Q plot for GWAS on ECT-treated MDD or TRD risk (ECT-treated MDD or TRD vs. healthy controls) in Sweden.

A) Mirrored Manhattan plots of GWAS on ECT-treated MDD or TRD risk in Sweden with ECT-treated MDD vs. healthy controls on the top and TRD vs. healthy controls on the bottom. The red line indicates genome-wide significance at $P<5.0\times10^{-8}$; the blue line indicates suggestive genome-wide significance at $P<5.0\times10^{-5}$.

B) Q-Q plot for GWAS on ECT-treated MDD risk.

C) Q-Q plot for GWAS on TRD risk.

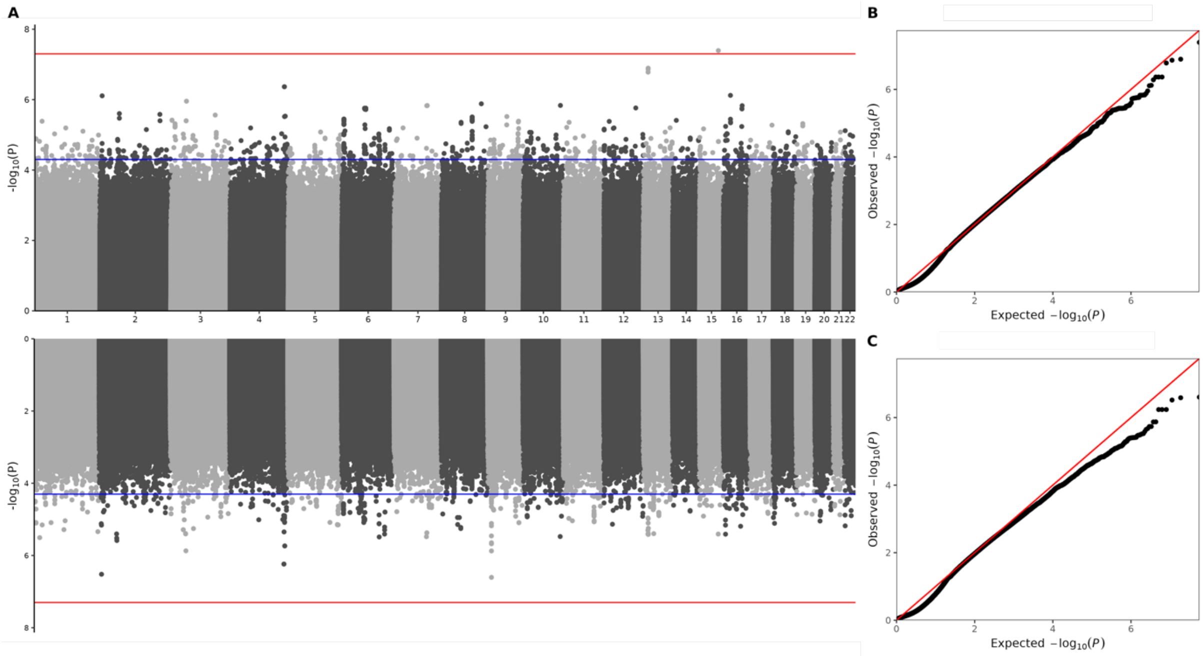

Supplementary Figure 3. Manhattan plot and Q-Q plot for GWAS on ECT-treated MDD or TRD risk (ECT-treated MDD or TRD vs. healthy controls) in Estonia Biobank.

A) Mirrored Manhattan plots of GWAS on ECT-treated MDD or TRD risk in Estonia Biobank with ECT-treated MDD vs. healthy controls on the top and TRD vs. healthy controls on the bottom. The red line indicates genome-wide significance at $P<5.0\times10^{-8}$; the blue line indicates suggestive genome-wide significance at $P<5.0\times10^{-5}$.

B) Q-Q plot for GWAS on ECT-treated MDD risk.

C) Q-Q plot for GWAS on TRD risk.

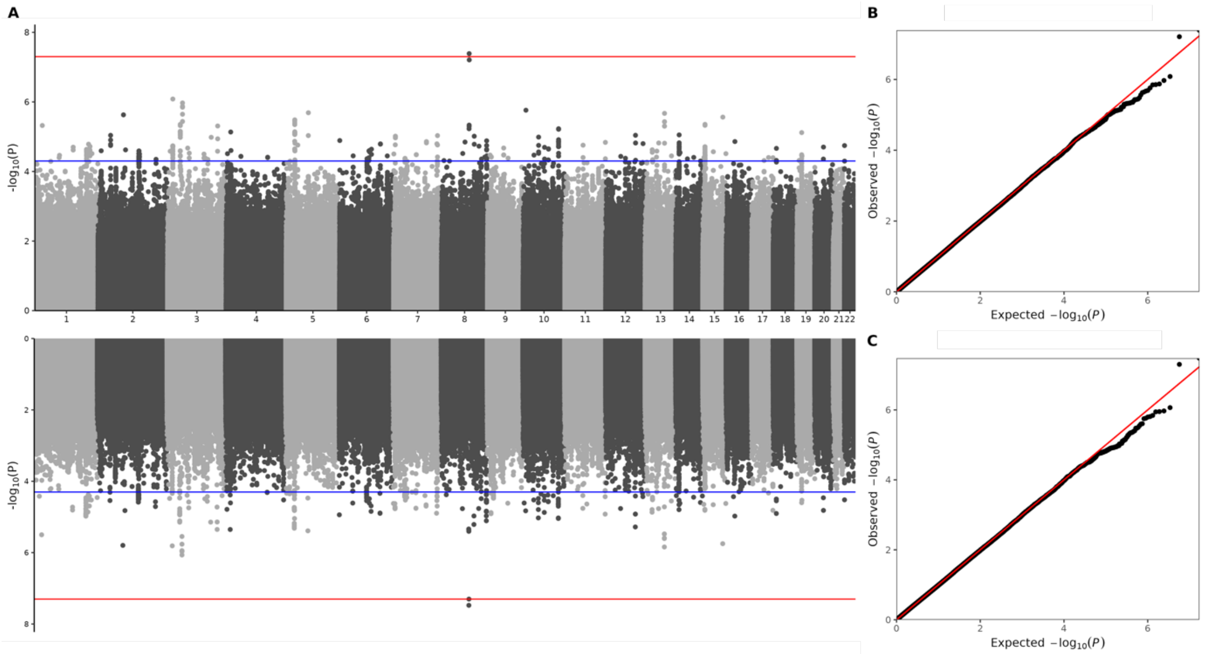

Supplementary Figure 4. Manhattan plot and Q-Q plot for GWAS on ECT-treated MDD or TRD risk (ECT-treated MDD or TRD vs. healthy controls) in FinnGen.

A) Mirrored Manhattan plots of GWAS on ECT-treated MDD or TRD risk in FinnGen with ECT-treated MDD vs. heathy controls on the top and TRD vs. heathy controls on the bottom. The red line indicates genome-wide significance at $P<5.0\times10^{-8}$; the blue line indicates suggestive genome-wide significance at $P<5.0\times10^{-5}$.

B) Q-Q plot for GWAS on ECT-treated MDD risk.

C) Q-Q plot for GWAS on TRD risk.

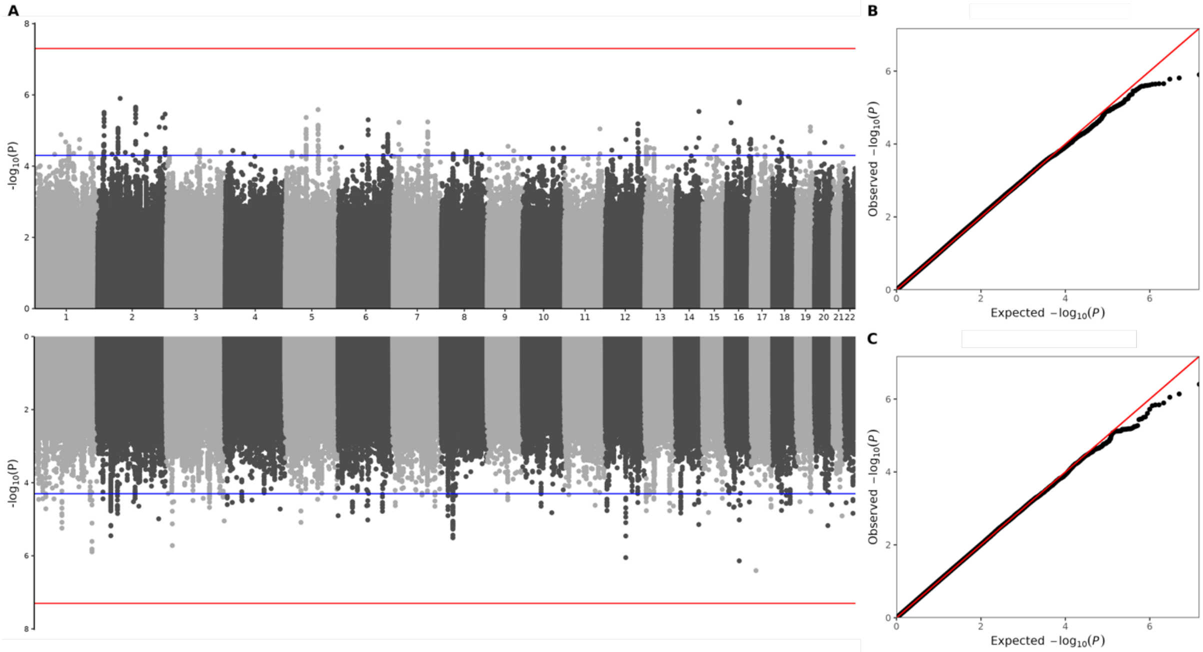

Supplementary Figure 5. Manhattan plot and Q-Q plot for GWAS on treatment resistance in MDD (TRD vs. non-TRD and ECT-treated MDD vs. non-ECT-treated MDD) in Sweden.

A) Mirrored Manhattan plots of GWAS on treatment resistance in MDD in Sweden with ECT-treated MDD vs. non-ECT-treated MDD on the top and TRD vs. non-TRD on the bottom. The red line indicates genome-wide significance at $P<5.0\times10^{-8}$; the blue line indicates suggestive genome-wide significance at $P<5.0\times10^{-5}$.

B) Q-Q plot for GWAS on ECT-treated MDD vs. non-ECT-treated MDD.

C) Q-Q plot for GWAS on TRD vs. non-TRD.

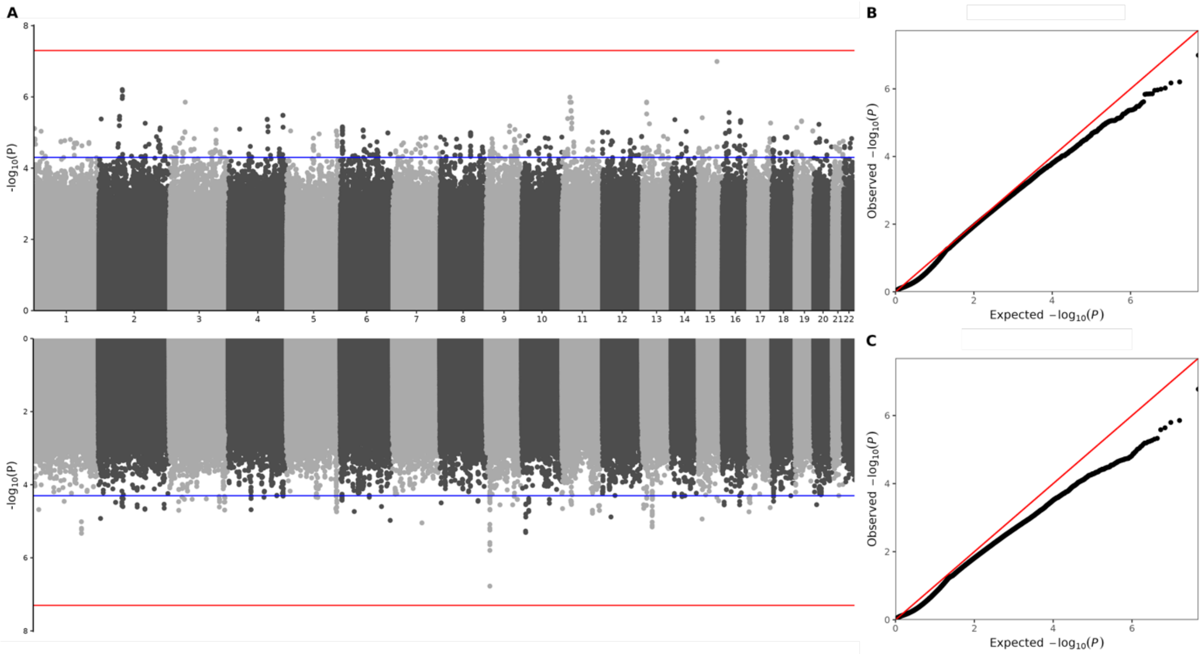

Supplementary Figure 6. Manhattan plot and Q-Q plot for GWAS on treatment resistance in MDD (TRD vs. non-TRD and ECT-treated MDD vs. non-ECT-treated MDD) in Estonia Biobank.

A) Mirrored Manhattan plots of GWAS on treatment resistance in MDD in Estonia Biobank with ECT-treated MDD vs. non-ECT-treated MDD on the top and TRD vs. non-TRD on the bottom. The red line indicates genome-wide significance at $P<5.0\times10^{-8}$; the blue line indicates suggestive genome-wide significance at $P<5.0\times10^{-5}$.

B) Q-Q plot for GWAS on ECT-treated MDD vs. non-ECT-treated MDD.

C) Q-Q plot for GWAS on TRD vs. non-TRD.

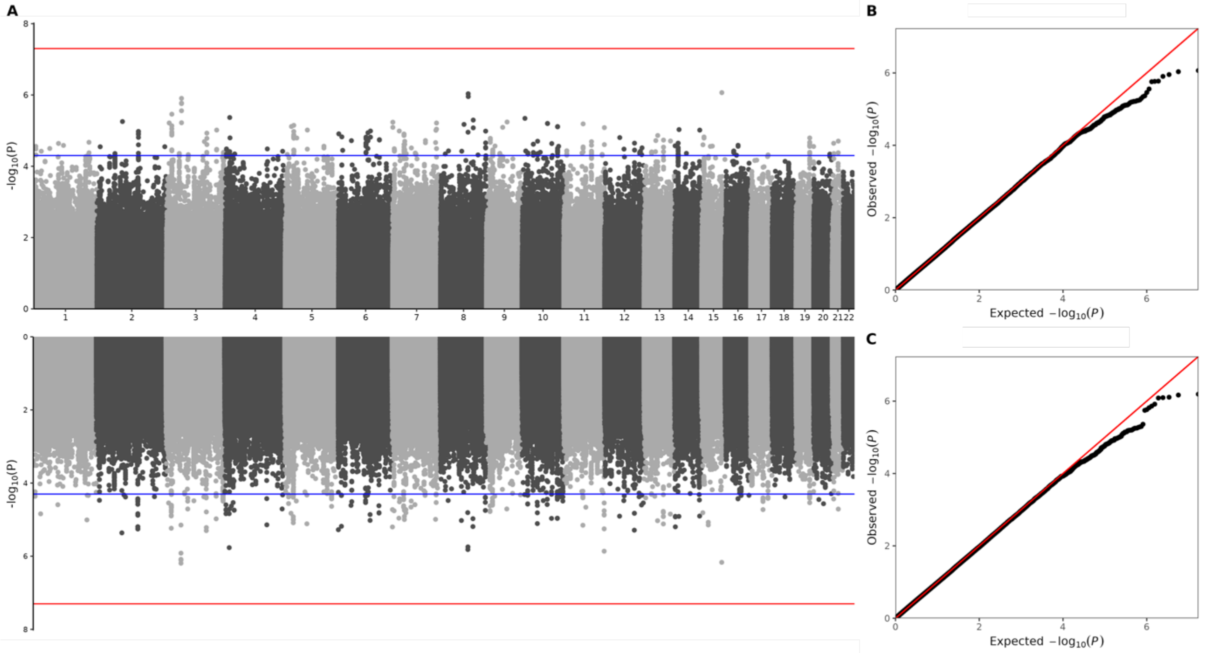

Supplementary Figure 7. Manhattan plot and Q-Q plot for GWAS on treatment resistance in MDD (TRD vs. non-TRD and ECT-treated MDD vs. non-ECT-treated MDD) in FinnGen.

A) Mirrored Manhattan plots of GWAS on treatment resistance in MDD in FinnGen with ECT-treated MDD vs. non-ECT-treated MDD on the top and TRD vs. non-TRD on the bottom. The red line indicates genome-wide significance at $P<5.0\times10^{-8}$; the blue line indicates suggestive genome-wide significance at $P<5.0\times10^{-5}$.

B) Q-Q plot for GWAS on ECT-treated MDD vs. non-ECT-treated MDD.

C) Q-Q plot for GWAS on TRD vs. non-TRD.

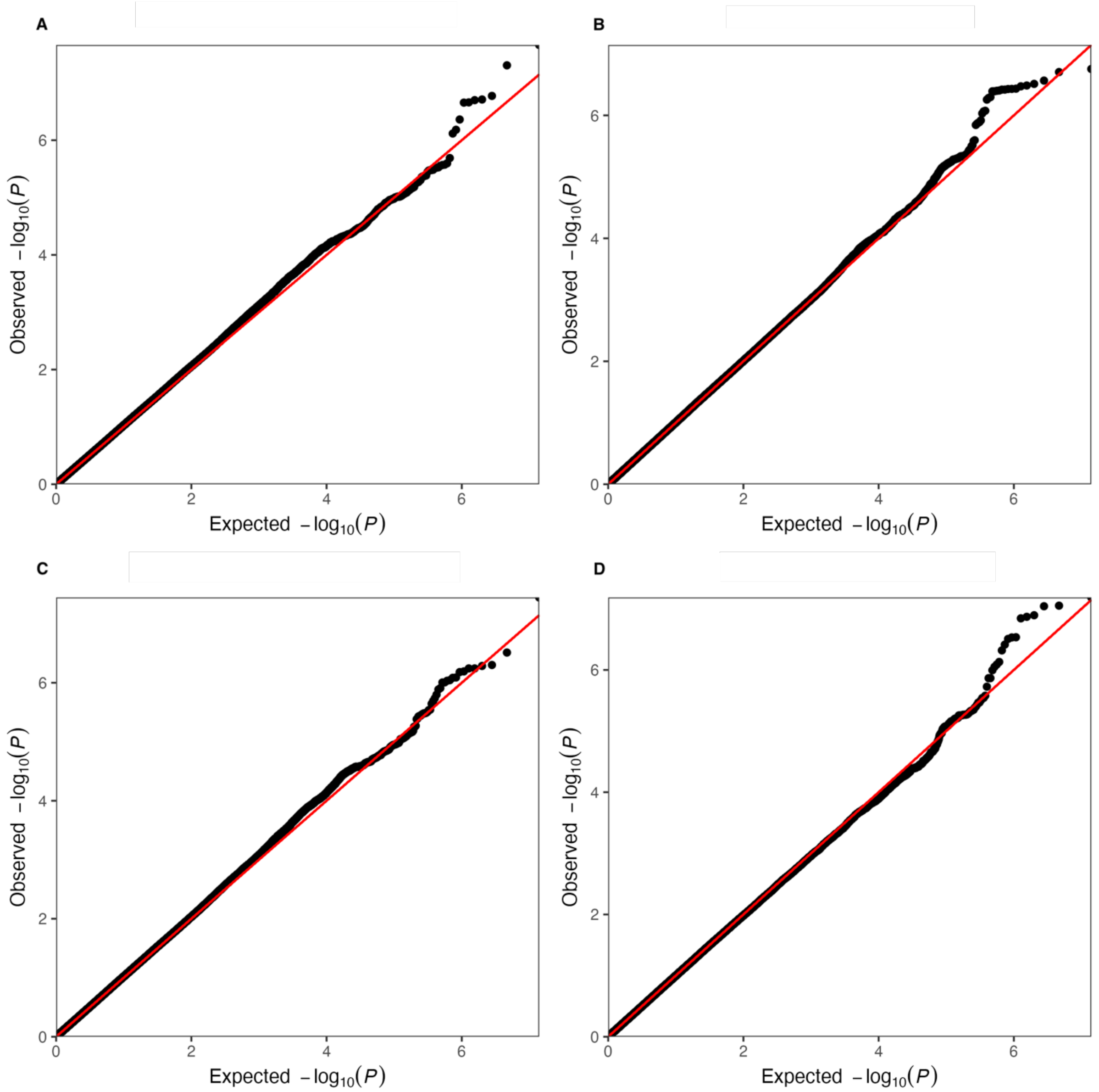

Supplementary Figure 8. Q-Q plot for GWAS meta-analysis on ECT-treated MDD/TRD risk (TRD vs. healthy controls) and treatment resistance in MDD (ECT-treated MDD vs. non-ECT-treated MDD and TRD vs. non-TRD). Q-Q plot of GWAS meta-analysis under different groups.

A) ECT-treated MDD vs. healthy controls

B) ECT-treated MDD vs. non-ECT-treated MDD

C) TRD vs. healthy controls

D) TRD vs. non-TRD

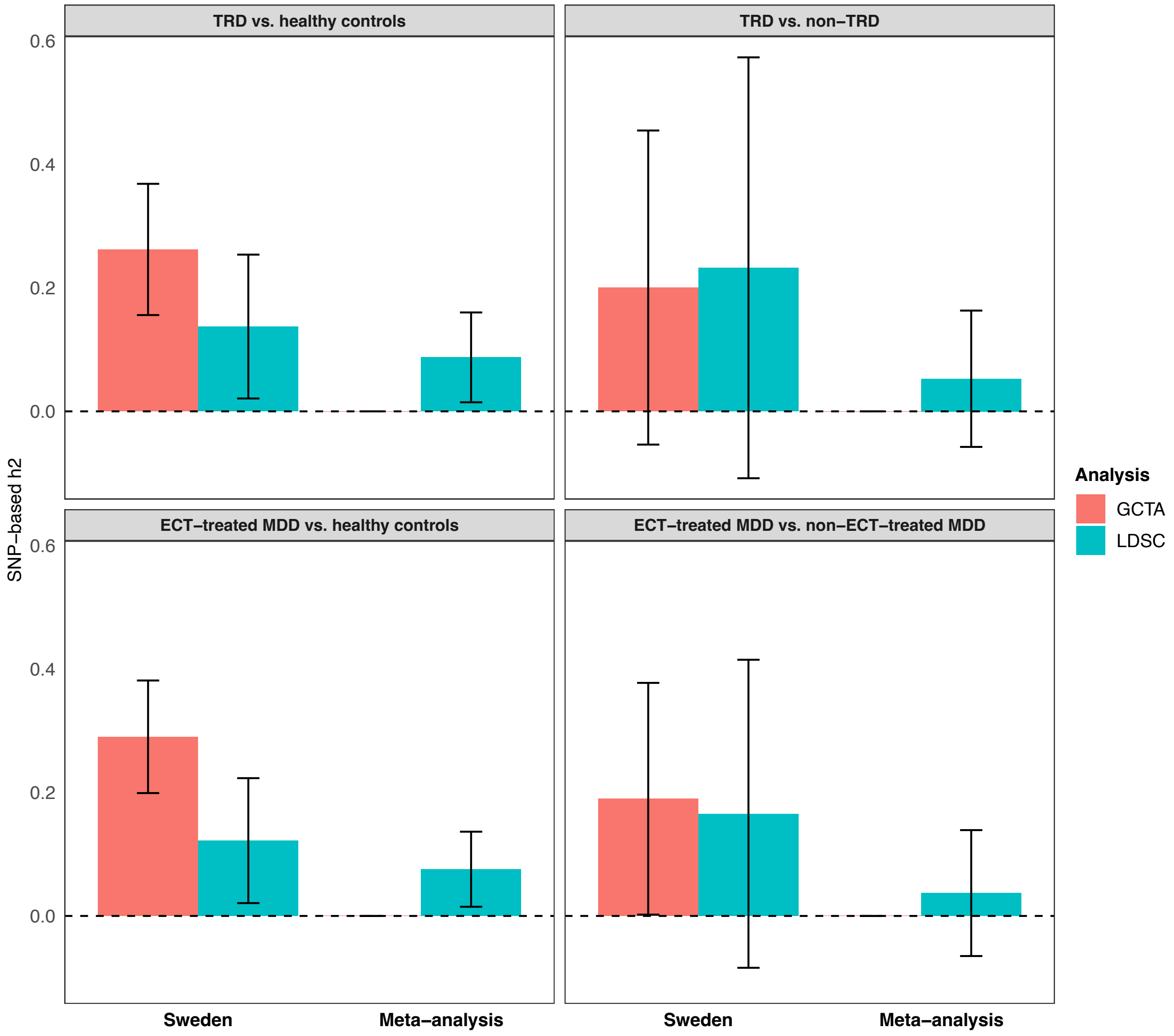

Supplementary Figure 9. Estimates of h^2_{SNP} on the liability scale using LDSC. The plot of h^2_{SNP} on the liability scale using LDSC and GCTA. Colours represent the sources of h^2_{SNP} estimates. To note, it needs caution to interpret the results of h^2_{SNP} due to the limited sample size, especially the results from LDSC as it shows good performance with large sample sizes. We reported estimates from: 1) Sweden – GCTA: Estimated using individual genotype data from the Swedish cohort in GCTA; 2) Sweden – LDSC: Estimated using summary statistics data from the Swedish cohort in LDSC; 3) GWAS meta-analysis LDSC: Estimated using summary statistics data from the GWAS meta-analysis in LDSC.

A.

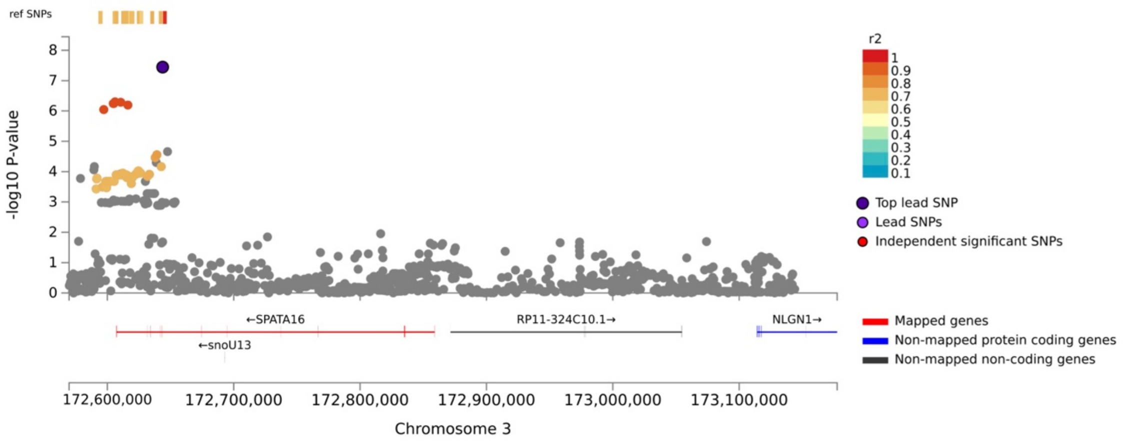

B.

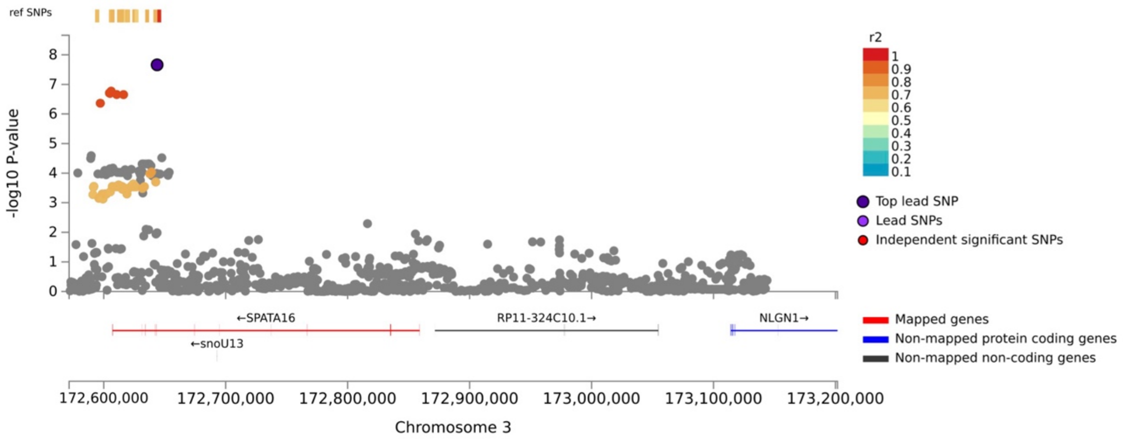

Supplementary Figure 10. Regional plots for top loci

A) Top locus from GWAS on TRD risk (TRD vs. healthy controls)

B) Top locus from GWAS on ECT-treated MDD risk (ECT-treated MDD vs. healthy controls)

C.

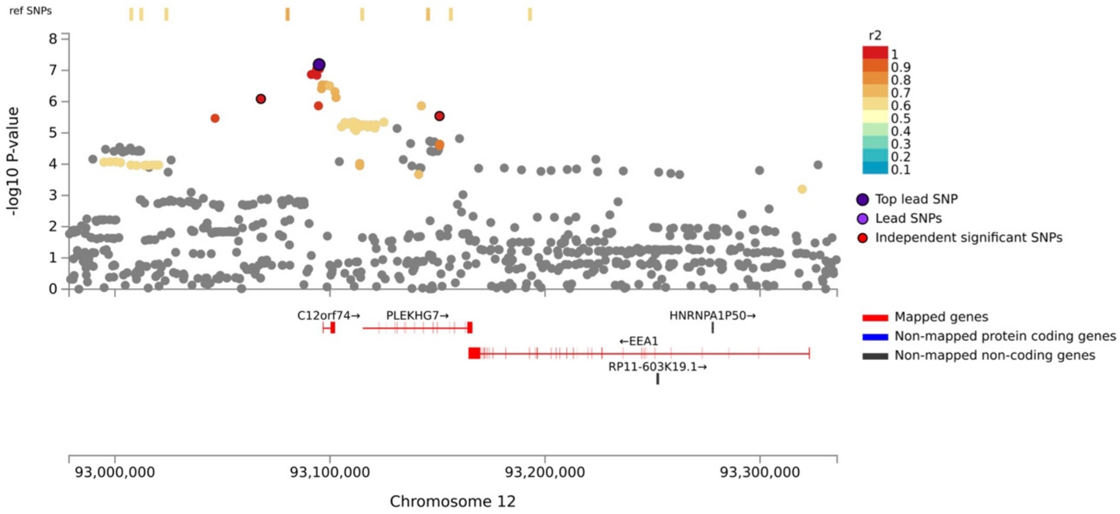

D.

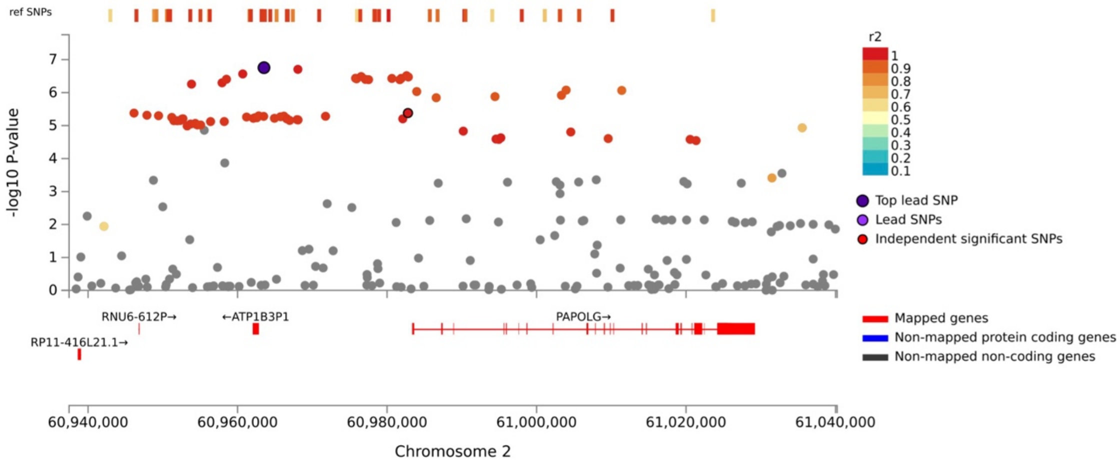

Supplementary Figure 10 (cont.). Regional plots for top loci

C) Top locus from GWAS on treatment resistance in MDD (TRD vs. non-TRD)

D) Top locus from GWAS on ECT-treated MDD vs. non-ECT-treated MDD

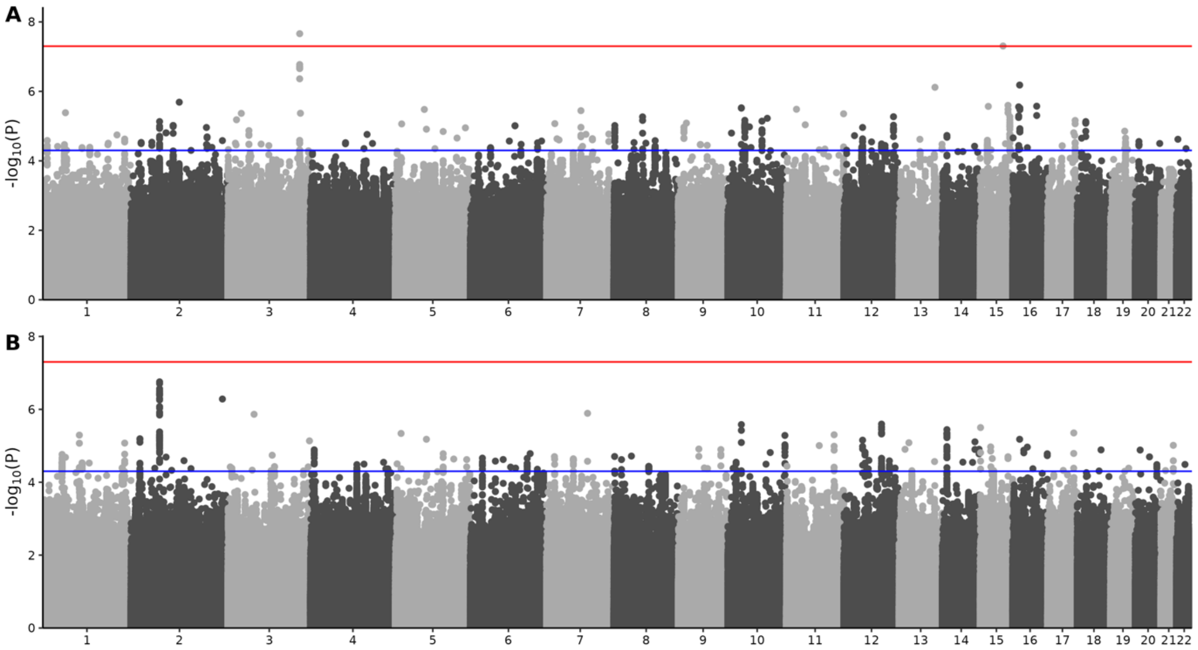

Supplementary Figure 11. Manhattan plots of GWAS meta-analysis

A) Manhattan plot of GWAS meta-analysis of ECT-treated MDD vs. healthy controls.

B) Manhattan plot of GWAS meta-analysis of ECT-treated MDD vs. non-ECT-treated MDD. The red line indicates genome-wide significance at $P<5.0\times10^{-8}$; the blue line indicates suggestive genome-wide significance at $P<5.0\times10^{-5}$.

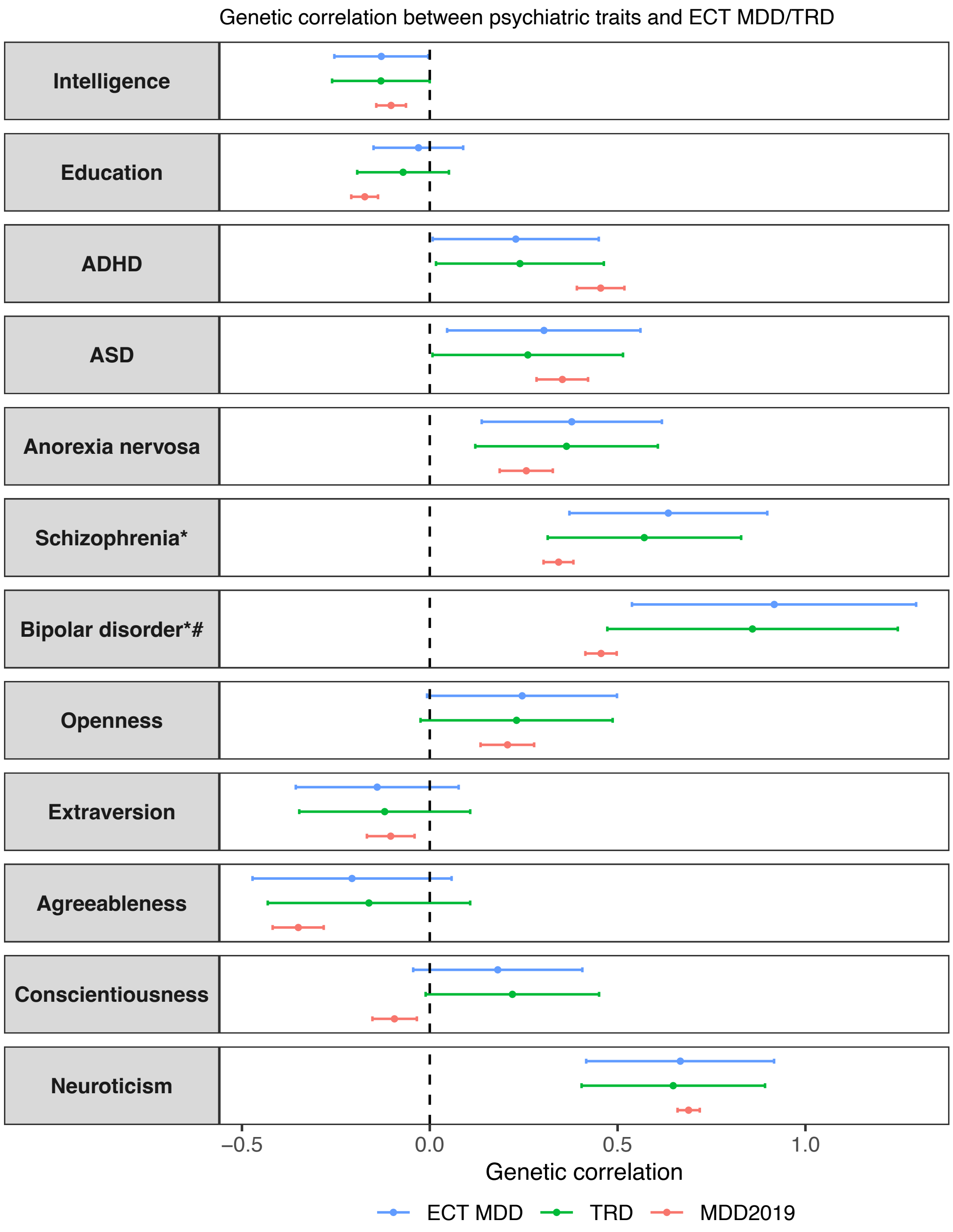

Supplementary Figure 12. Forest plot of genetic correlation between ECT-treated MDD or TRD risk and other psychiatric or cognitive traits.

*: significant difference in r_g between ECT-treated MDD vs. healthy controls and other psychiatric traits compared to r_g between MDD and other psychiatric traits.

#: significant difference in r_g between TRD vs. healthy controls and other psychiatric traits compared to r_g between MDD and other psychiatric traits. The statistical test is a two-sample z-test.

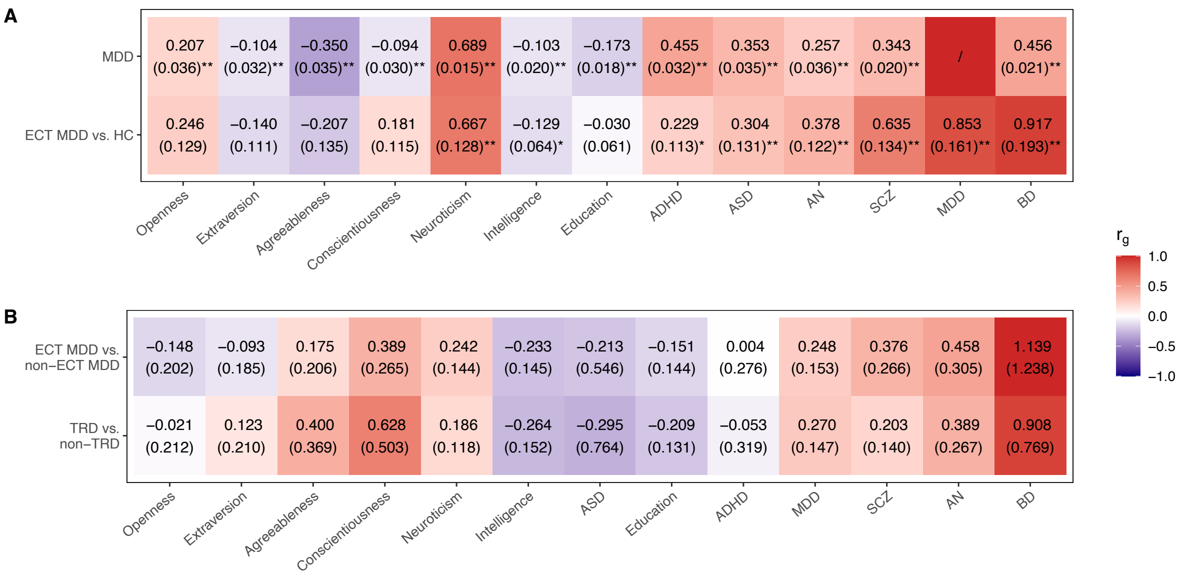

Supplementary Figure 13. Genetic correlations (r_g) of ECT-treated MDD risk and treatment resistance in MDD with other psychiatric disorders and cognitive traits. The plot is ordered by the r_g of MDD and other psychiatric disorders and cognitive traits, with personality traits grouped together. ADHD: attention deficit hyperactivity disorder; AN: anorexia nervosa; ASD: autism spectrum disorder; BD: bipolar disorders; MDD: major depressive disorder; SCZ: schizophrenia.

A) R_g of ECT-treated MDD risk (ECT-treated MDD vs. healthy controls) and other psychiatric disorders and cognitive traits

B) R_g of treatment resistance in MDD (TRD vs. non-TRD or ECT-treated MDD vs. non-ECT-treated MDD) and other psychiatric disorders and cognitive traits. *Rg*>1 for ECT MDD vs. non-ECT MDD and bipolar disorder was due to sampling variation which is likely to be caused by lowered sample size in the summary statistics from case-only GWAS (within MDD cases).

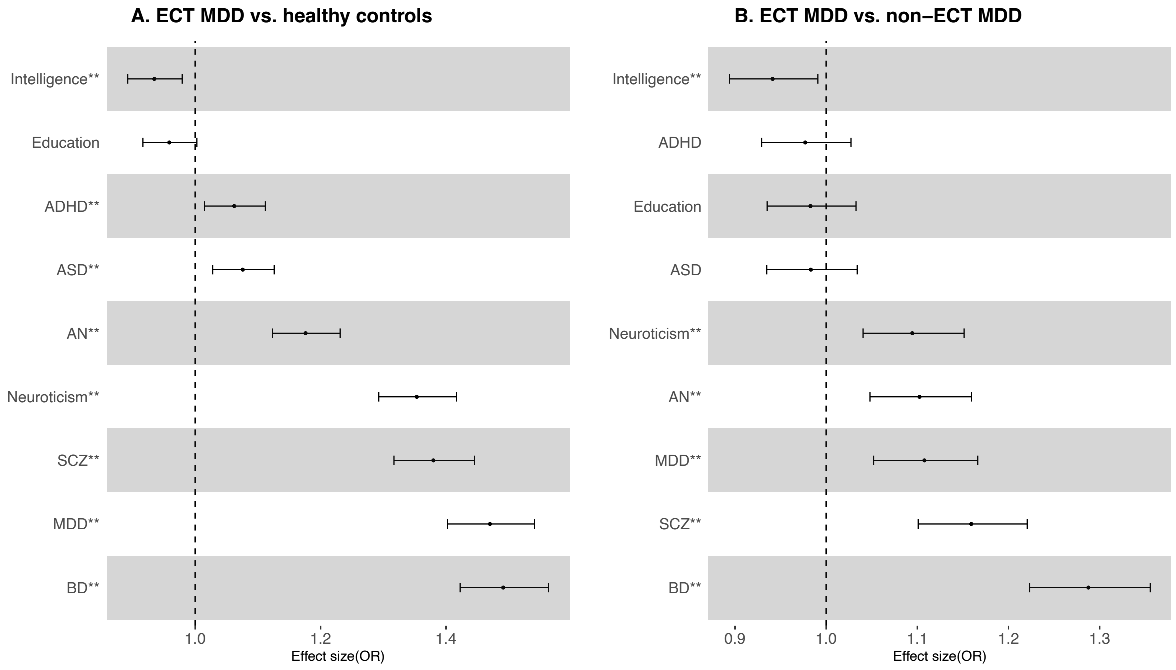

Supplementary Figure 14. Associations between PRS of other psychiatric disorders and cognitive traits and ECT-treated MDD. In both panels, the traits are ordered by the effect sizes. ADHD: attention deficit hyperactivity disorder; AN: anorexia nervosa; ASD: autism spectrum disorder; BD: bipolar disorders; MDD: major depressive disorder; SCZ: schizophrenia.

(A) The association between PRS of psychiatric and cognitive traits and ECT MDD risk (ECT-treated MDD compared to healthy controls). ORs for ECT-treated MDD are associated with each SD increase in the PRS of other traits.

(B) The association between PRS of psychiatric and cognitive traits and ECT-treated MDD compared to non-ECT-treated MDD.

The traits are ordered by the effect sizes. * Significance at nominal P<0.05; ** FDR<0.05.

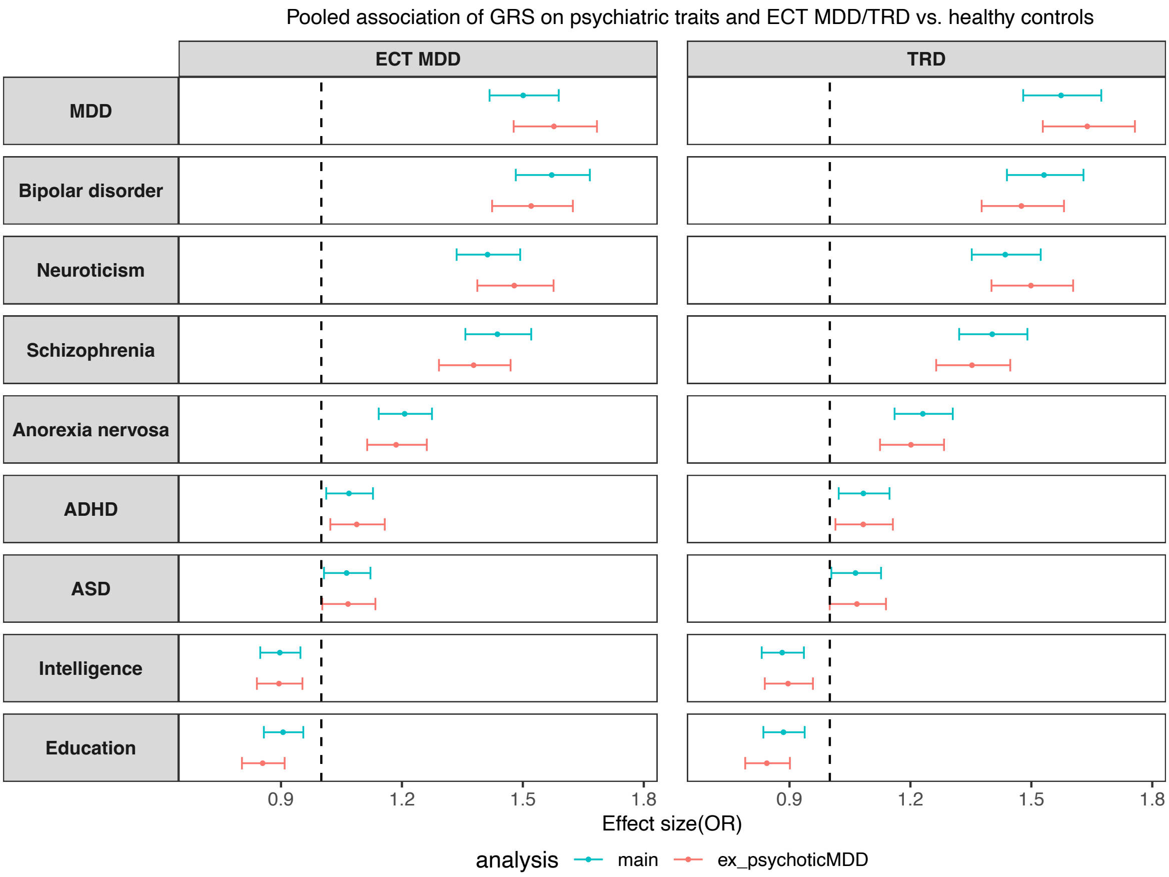

Supplementary Figure 15. The association between PRS of psychiatric traits and ECT-treated MDD/TRD risk excluding psychotic MDD in Swedish samples (case-control comparison). ORs for TRD are associated with each SD increase in the PRS of other psychiatric traits. The left panel is the association between PRS of psychiatric traits and ECT-treated MDD vs. healthy controls, and the right panel is the association between PRS of psychiatric traits and TRD risk.

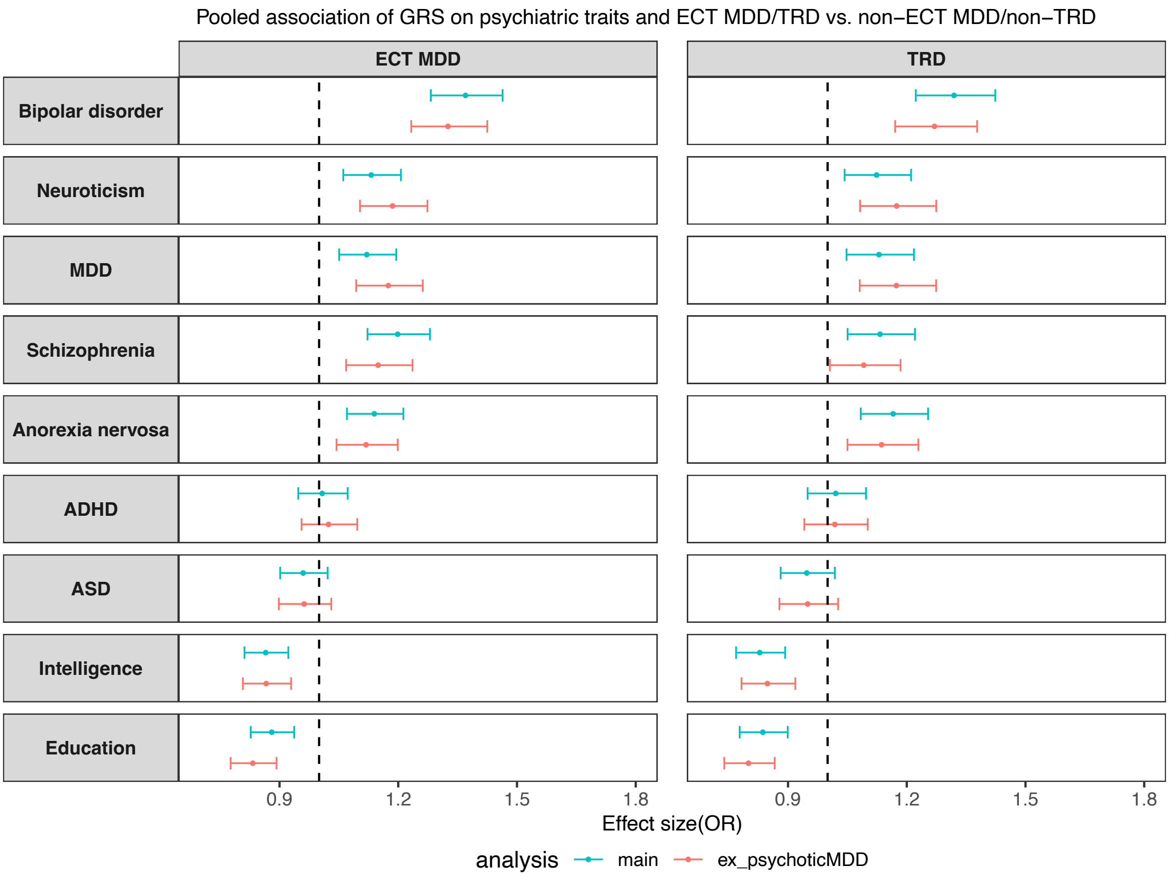

Supplementary Figure 16. The association between PRS of psychiatric traits and treatment resistance in MDD excluding psychotic MDD in Swedish samples (case-case comparison). ORs for TRD are associated with each SD increase in the PRS of other psychiatric traits. The left panel is the association between PRS of psychiatric traits and ECT-treated MDD vs. non-ECT-treated MDD, and the right panel is the association between PRS of psychiatric traits and TRD vs. non-TRD.

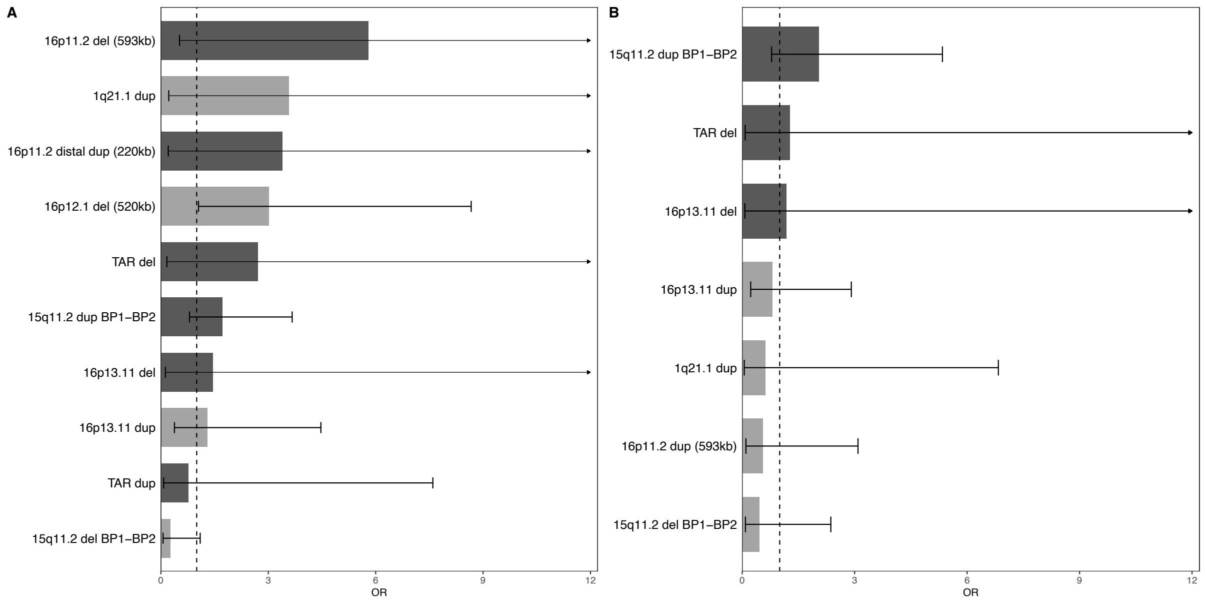

Supplementary Figure 17. Associations of known neuropsychiatric CNVs and ECT-treated MDD. The figure only showed those with CNV detected by both the case and the comparison groups. In both panels, the CNVs were ordered by the ORs. The analysis was done within the Swedish cohort.

(A) The association between neuropsychiatric CNV and ECT-treated MDD risk when comparing ECT MDD with healthy controls

(B) The association between neuropsychiatric CNV and ECT-treated MDD compared to non-ECT-treated MDD

References

1. Clements CC, Karlsson R, Lu Y, Juréus A, Rück C, Andersson E, et al. Genome-wide association study of patients with a severe major depressive episode treated with electroconvulsive therapy. Mol Psychiatry. 2021;26:2429–2439.
2. Andersson E, Crowley JJ, Lindefors N, Ljótsson B, Hedman-Lagerlöf E, Boberg J, et al. Genetics of response to cognitive behavior therapy in adults with major depression: a preliminary report. Mol Psychiatry. 2019;24:484–490.
3. Zagai U, Lichtenstein P, Pedersen NL, Magnusson PKE. The Swedish Twin Registry: Content and Management as a Research Infrastructure. Twin Res Hum Genet. 2019;22:672–680.
4. Xiong Y, Karlsson R, Song J, Kowalec K, Rück C, Sigström R, et al. Polygenic risk scores of lithium response and treatment resistance in major depressive disorder. Transl Psychiatry. 2023;13:301.
5. Lam M, Awasthi S, Watson HJ, Goldstein J, Panagiotaropoulou G, Trubetskoy V, et al. RICOPILI: Rapid Imputation for COnsortias PIpeLIne. Bioinformatics. 2020;36:930–933.
6. McCarthy S, Das S, Kretzschmar W, Delaneau O, Wood AR, Teumer A, et al. A reference panel of 64,976 haplotypes for genotype imputation. Nat Genet. 2016;48:1279–1283.
7. Loh P-R, Danecek P, Palamara PF, Fuchsberger C, A Reshef Y, K Finucane H, et al. Reference-based phasing using the Haplotype Reference Consortium panel. Nat Genet. 2016;48:1443–1448.
8. Howie BN, Donnelly P, Marchini J. A flexible and accurate genotype imputation method for the next generation of genome-wide association studies. PLoS Genet. 2009;5:e1000529.
9. Durbin R. Efficient haplotype matching and storage using the positional Burrows-Wheeler transform (PBWT). Bioinformatics. 2014;30:1266–1272.
10. Leitsalu L, Haller T, Esko T, Tammesoo M-L, Alavere H, Snieder H, et al. Cohort Profile: Estonian Biobank of the Estonian Genome Center, University of Tartu. Int J Epidemiol. 2015;44:1137–1147.
11. Ojalo T, Haan E, Kõiv K, Kariis HM, Krebs K, Uusberg H, et al. Cohort Profile Update: Mental Health Online Survey in the Estonian Biobank (EstBB MHoS). Int J Epidemiol. 2024;53.
12. Browning SR, Browning BL. Rapid and accurate haplotype phasing and missing-data inference for whole-genome association studies by use of localized haplotype clustering. Am J Hum Genet. 2007;81:1084–1097.
13. Browning BL, Zhou Y, Browning SR. A One-Penny Imputed Genome from Next-Generation Reference Panels. Am J Hum Genet. 2018;103:338–348.
14. Mitt M, Kals M, Pärn K, Gabriel SB, Lander ES, Palotie A, et al. Improved imputation accuracy of rare and low-frequency variants using population-specific high-coverage WGS-based imputation reference panel. Eur J Hum Genet. 2017;25:869–876.
15. Mbatchou J, Barnard L, Backman J, Marcketta A, Kosmicki JA, Ziyatdinov A, et al. Computationally efficient whole-genome regression for quantitative and binary traits. Nat Genet. 2021;53:1097–1103.
16. Kurki MI, Karjalainen J, Palta P, Sipilä TP, Kristiansson K, Donner KM, et al. FinnGen provides genetic insights from a well-phenotyped isolated population. Nature. 2023;613:508–518.
17. Huang J, Howie B, McCarthy S, Memari Y, Walter K, Min JL, et al. Improved imputation of low-frequency and rare variants using the UK10K haplotype reference panel. Nat Commun. 2015;6:8111.
18. Bellenguez C, Strange A, Freeman C, Wellcome Trust Case Control Consortium, Donnelly P, Spencer CCA. A robust clustering algorithm for identifying problematic samples in genome-wide association studies. Bioinformatics. 2012;28:134–135.
19. Zhou W, Nielsen JB, Fritsche LG, Dey R, Gabrielsen ME, Wolford BN, et al. Efficiently controlling for case-control imbalance and sample relatedness in large-scale genetic association studies. Nat Genet. 2018;50:1335–1341.

20. Kellner CH, Greenberg RM, Murrough JW, Bryson EO, Briggs MC, Pasculli RM. ECT in treatment-resistant depression. Am J Psychiatry. 2012;169:1238–1244.
21. Lisanby SH. Electroconvulsive therapy for depression. N Engl J Med. 2007;357:1939–1945.
22. Baghai TC, Möller H-J. Electroconvulsive therapy and its different indications. Dialogues Clin Neurosci. 2008;10:105–117.
23. Fabbri C, Hagenaars SP, John C, Williams AT, Shrine N, Moles L, et al. Genetic and clinical characteristics of treatment-resistant depression using primary care records in two UK cohorts. Mol Psychiatry. 2021;26:3363–3373.
24. Fava M. Diagnosis and definition of treatment-resistant depression. Biol Psychiatry. 2003;53:649–659.
25. Lagerberg T, Fazel S, Molero Y, Franko MA, Chen Q, Hellner C, et al. Associations between selective serotonin reuptake inhibitors and violent crime in adolescents, young, and older adults - a Swedish register-based study. Eur Neuropsychopharmacol. 2020;36:1–9.
26. Kang J, Castro VM, Ripperger M, Venkatesh S, Burstein D, Linnér RK, et al. Genome-Wide Association Study of Treatment-Resistant Depression: Shared Biology With Metabolic Traits. Am J Psychiatry. 2024:appiajp20230247.
27. Wellcome Trust Case Control Consortium, Craddock N, Hurles ME, Cardin N, Pearson RD, Plagnol V, et al. Genome-wide association study of CNVs in 16,000 cases of eight common diseases and 3,000 shared controls. Nature. 2010;464:713–720.
28. Szatkiewicz JP, O'Dushlaine C, Chen G, Chambert K, Moran JL, Neale BM, et al. Copy number variation in schizophrenia in Sweden. Mol Psychiatry. 2014;19:762–773.
29. Watanabe K, Taskesen E, van Bochoven A, Posthuma D. Functional mapping and annotation of genetic associations with FUMA. Nat Commun. 2017;8:1826.
30. Boughton AP, Welch RP, Flickinger M, VandeHaar P, Taliun D, Abecasis GR, et al. LocusZoom.js: interactive and embeddable visualization of genetic association study results. Bioinformatics. 2021;37:3017–3018.
31. Lloyd-Jones LR, Zeng J, Sidorenko J, Yengo L, Moser G, Kemper KE, et al. Improved polygenic prediction by Bayesian multiple regression on summary statistics. Nat Commun. 2019;10:5086.
32. Ni G, Zeng J, Revez JA, Wang Y, Zheng Z, Ge T, et al. A Comparison of Ten Polygenic Score Methods for Psychiatric Disorders Applied Across Multiple Cohorts. Biol Psychiatry. 2021;90:611–620.
33. Purcell S, Neale B, Todd-Brown K, Thomas L, Ferreira MAR, Bender D, et al. PLINK: a tool set for whole-genome association and population-based linkage analyses. Am J Hum Genet. 2007;81:559–575.
